# Mining of *Brassica*-Specific Genes (BSGs) and Their Induction in Different Developmental Stages and under *Plasmodiophora brassicae* Stress in *Brassica rapa*

**DOI:** 10.3390/ijms19072064

**Published:** 2018-07-16

**Authors:** Mingliang Jiang, Xiangshu Dong, Hong Lang, Wenxing Pang, Zongxiang Zhan, Xiaonan Li, Zhongyun Piao

**Affiliations:** 1 College of Horticulture, Shenyang Agricultural University, #120 Dongling Road, Shenyang 110866, China; dr_jiang@syau.edu.cn (M.J.); pwxsyau@163.com (W.P.); zhanzxiang@126.com (Z.Z.); gracesleexn@163.com (X.L.); 2 School of Agriculture, Yunnan University, Kunming 650504, China; dongxiangshu@ynu.edu.cn; 3 Key Laboratory of Northeast Rice Biology and Breeding, Ministry of Agriculture, Rice Research Institute, Shenyang Agricultural University, Shenyang 110866, China; lang_0916@163.com

**Keywords:** *Brassica*-specific genes, identification, validation, gene expression, *Plasmodiophora brassicae*

## Abstract

Orphan genes, also called lineage-specific genes (LSGs), are important for responses to biotic and abiotic stresses, and are associated with lineage-specific structures and biological functions. To date, there have been no studies investigating gene number, gene features, or gene expression patterns of orphan genes in *Brassica rapa*. In this study, 1540 *Brassica*-specific genes (BSGs) and 1824 Cruciferae-specific genes (CSGs) were identified based on the genome of *Brassica rapa*. The genic features analysis indicated that BSGs and CSGs possessed a lower percentage of multi-exon genes, higher GC content, and shorter gene length than evolutionary-conserved genes (ECGs). In addition, five types of BSGs were obtained and 145 out of 529 real A subgenome-specific BSGs were verified by PCR in 51 species. In silico and semi-qPCR, gene expression analysis of BSGs suggested that BSGs are expressed in various tissue and can be induced by *Plasmodiophora brassicae*. Moreover, an A/C subgenome-specific BSG, *BSGs1*, was specifically expressed during the heading stage, indicating that the gene might be associated with leafy head formation. Our results provide valuable biological information for studying the molecular function of BSGs for *Brassica*-specific phenotypes and biotic stress in *B. rapa*.

## 1. Introduction

Orphan genes are genes that are unique to a particular taxon, which exhibit no significant sequence similarity outside their species. These genes are also known as taxonomically restricted genes (TRGs) or lineage-specific genes (LSGs), and they are also commonly referred to as “ORFans” (opening reading frames (ORFs) with no detectable sequence similarity to any other ORF in a targeted database) [1,2,3,4]. With the development of sequencing technologies and genome analysis methods, research has shown that orphan genes are common in all kingdoms of life [3,5], and orphan genes represent 5–15% of the genomes of several species [1,5]. Orphan genes can be determined by comparative genomic analysis across various species using BLAST programs [5]. The identification and characterization of orphan genes have been analyzed in several organisms, including yeast [6], rice [7], *Arabidopsis* [3,8,9,10], and sweet orange [11]. Further, no study of orphan genes in *Brassica rapa* has been reported until now.

Despite the wildly found orphans in kingdoms of life, only a small number of them have been well characterized as a result of the absence of functional motifs, identifiable folds, and recognizable domains. Qua-quine starch (*QQS*; TAIR: *AT3G30720*) is the first plant orphan gene with a biochemically characterized function, and it is known to regulate carbon and nitrogen partitioning to lipid, starch, and protein in seeds and leaves across species through NF-YC (a component of the nuclear factor Y complex, is conserved across eukaryotes) interactions [5,12,13,14]. A wheat *Triticum aestivum Fusarium* resistance orphan gene (*TaFROG*) contributes to disease resistance in wheat [15]. The Brassicaceae-specific *Enhancer of vascular Wilt Resistance 1* (*EWR1*) in *Arabidopsis* and *Brassica oleracea* is responsible for the enhancement of resistance to *Verticillium* wilt pathogens [16]. Gene profiling and mutant screening also provide some clues to the functions of orphans. In *Arabidopsis*, rice, and sweet orange, the results of mutant screening or qRT-PCR analyses indicated that orphan genes were associated with biotic and abiotic stress responses [10,11,17]. The transcriptome of moss *Physcomitrella patens* indicated that early cold stress responses were dominated by orphan genes [2]. The specialized structures for bacterial symbiosis in legumes were controlled by family-specific secreted, proline-rich tandem repeat proteins (TRPs) [18]. The secreted orphan genes were highly enriched by aphid-specific cells by deep sequencing in *Buchnera aphidicola* [19]. Given all that, orphan genes are likely to be associated with abiotic or biotic stress responses and species-specific adaptive processes or traits [5,16,17,20].

Cruciferae is one of the most diverse plant families, containing over 3660 species, including economically important edible and industrial oilseed and vegetable crops [21]. The genus *Brassica* is one of 51 genera in the Cruciferae family, and it is comprised of several valuable crops that are used for human nutrition and that provide important insights into genome evolution research [22,23]. Cultivated *Brassica* species include six *Brassica* crops described by the U’s triangle [24]. These crops include diploid *Brassica rapa* (A genome), *Brassica nigra* (B genome), and *Brassica oleracea* (C genome), as well as allotetraploid *Brassica juncea* (AB genomes), *Brassica napus* (AC genomes), and *Brassica carinata* (BC genomes) [23,25]. *Brassica* species display extreme morphological characteristics resulting from artificial selection during domestication and breeding, such as leafy heads, enlarged organs, and extensive axillary branching [26]. Leaf-heading traits are agronomic characteristics with great economic and breeding values that directly influence crop yield and marketability, which often attracts the attention of breeders [27]. Several studies have focused on determining how several factors such as light intensity, temperature, carbohydrate to nitrogen ratios, and auxin concentrations affect the leafy head formation [28]. Furthermore, *Plasmodiophora brassicae* is an obligate parasite and soil-borne biotrophic pathogen of *Brassica* crops and other Cruciferae species, leading to clubroot and subsequent reduction in crop yield [29,30,31]. Therefore, it is beneficial to investigate the interactions between *P. brassicae* and Cruciferae species, because the results may offer a theoretical basis for the breeding of resistance in Cruciferae species. Because of clues of orphan genes provided by previous reports, we assumed that the *Brasssica*-specific genes may associate with the various morphological characteristics of *Brassica* species and the response to *P. brassicae*.

In this study, we identified and characterized *Brassica*-specific genes (BSGs), Cruciferae-specific genes (CSGs), and evolutionary-conserved genes (ECGs) using the genome of *B. rapa*, and a more stringent homologous sequence searching criteria was used than in previous comparative analyses of orphan genes. To explore the BSGs functions, the expression patterns of BSGs in various tissues and their responsive patterns to *P. brassicae* were analyzed using previously published RNA sequencing data [32,33] and semi-qPCR in *B. rapa*.

## 2. Results

### 2.1. Identification of BSGs and CSGs in B. rapa

**Figure 1:**
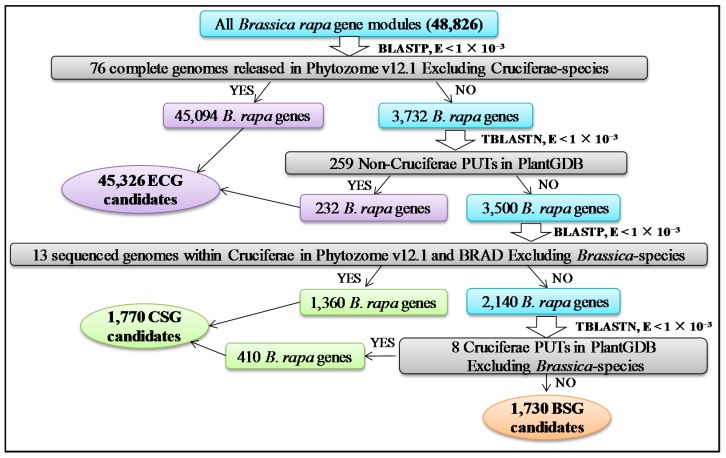
The procedure to identify candidate *Brassica*-specific genes (BSGs), Cruciferae-specific genes (CSGs), and evolutionary-conserved genes (ECGs) in *B. rapa* genome. Gray boxes indicated plant genomes and plant genome database (PlantGDB) generated unique transcripts (PUTs) excluding *Brassica rapa*. Blue boxes represented the *B. rapa* genes. Purple boxes indicated *B. rapa* genes belonging to ECG candidates. Green boxes and orange boxes indicated *B. rapa* genes belonging to CSG and BSG candidates, respectively. BRAD—*Brassica* database.

With the recent availability of several plant genomes and transcriptome sequences, an upgraded method was used to identify ECGs, CSGs, and BSGs, compared with that used in former studies [9,11,34,35,36]. A total of 48,826 *B. rapa* gene modules were compared with 76 non-Cruciferae genome sequences that were released in Phytozome v12.1 using BLASTP. A total of 45,094 *B. rapa* genes with significant sequence similarity (*E*-value < 1 × 10^−3^) to at least one sequence from a species outside the Cruciferae family were defined as ECG candidates, and were subsequently eliminated from further analysis (Figure 1). The remaining 3732 *B. rapa* genes with no significant similarity to each non-Cruciferae genome were compared with PlantGDB-generated Unique Transcripts (PUTs) from 259 non-Cruciferae species using TBLASTN. Moreover, 232 *B. rapa* genes with significant similarity (*E*-value < 1 × 10^−3^) to at least one non-Cruciferae PUT were classified as ECGs. The remaining 3500 *B. rapa* genes with no significant similarity were further compared with 13 Cruciferae genomes (excluding *Brassica* species) using BLASTP. A total of 1360 *B. rapa* genes showed significant similarity (*E*-value < 1 × 10^−3^) with at least one sequence of Cruciferae family species (excluding *Brassica* species), and were defined as CSG candidates before removal from further analysis (Figure 1). The remaining 2140 *B. rapa* genes without homologs in non-Cruciferae genome were used for further analysis, which compared the genes with PUTs from eight Cruciferae species (excluding *Brassica* species) using TBLASTN. A total of 410 *B. rapa* genes with significant similarity (*E*-value < 1 × 10^−3^) to at least one Cruciferae PUT were classified as CSG candidates. The remaining 1730 *B. rapa* genes without homologs in Cruciferae genomic sequences or PUTs (excluding *Brassica* species) were defined as BSG candidates (Figure 1).

**Figure 2:**
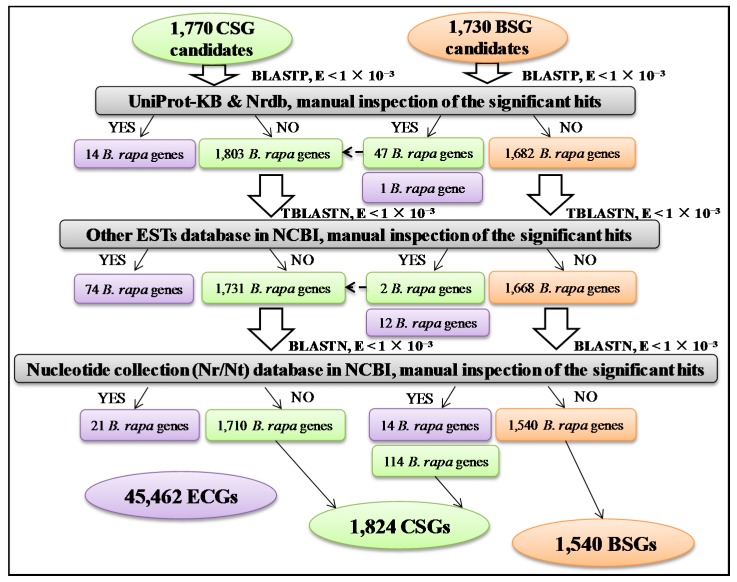
Procedure for identifying BSGs, CSGs, and ECGs in *B. rapa* genomes. UniProt-KB—UniProt Knowledgebase; Nrdb—non-redundant protein database; ESTs: expressed sequence tags; NCBI: national center for biotechnology information.

In order to further eliminate false positives resulting from incomplete genomes, BSG and CSG candidates were compared with the UniProt Knowledgebase (UniProt-KB) and the non-redundant protein database (Nrdb) using BLASTP (Figure 2). After manual inspection of the alignments (*E*-value < 1 × 10^−3^), 15 genes (14 CSGs and one BSG) were defined as ECGs. Furthermore, 47 BSGs with significant similarity to at least one Cruciferae sequence (excluding *Brassica* species) in UniProt-KB or Nrdb were classified as CSGs. Furthermore, 1682 BSG and 1803 CSG candidates were compared with other expressed sequence tags (ESTs) database in national center for biotechnology information (NCBI) using TBLASTN. After manual inspection of the alignments (*E*-value < 1 × 10^−3^; query cover and identity <70%), 86 genes (74 CSGs and 12 BSGs) were classified as ECGs. Additionally, two BSGs with significant similarity (*E*-value < 1 × 10^−3^; query cover and identity ≥70%) to at least one EST sequence within Cruciferae (excluding *Brassica* species) in NCBI were classified as CSGs. The coding-sequences of 1668 remaining BSG and 1731 CSG candidates were compared with the Nucleotide collection (Nr/Nt) database in NCBI using BLASTN. Manual inspection of the alignments (*E*-value < 1 × 10^−3^; query cover and identity <70%) identified 35 genes (21 CSGs and 14 BSGs) with sequence similarity (*E*-value < 1 × 10^−3^; query cover and identity ≥70%) to non-Cruciferae sequences, which were classified as ECGs. Furthermore, 114 BSGs were designated as CSGs, and the final BSGs, CSGs, and ECGs sets contained 1540, 1824, and 45,462 *B. rapa* genes, respectively (Figure 2).

### 2.2. Genic Features of the BSGs and CSGs in B. rapa

**Figure 3:**
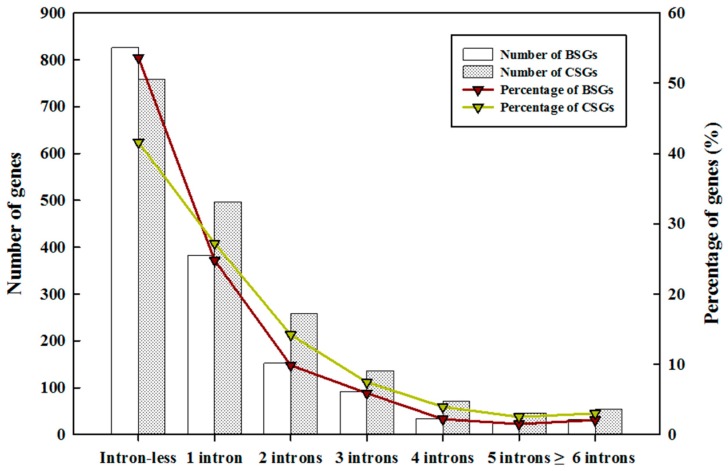
Intron numbers analysis of BSGs and CSGs. Gene structures were divided into seven types, both numbers and percentages of different types of intron number are shown. The *X*-axis indicates seven types of intron number. The left-*Y*-axis represents the number of different types of intron numbers of BSGs (blank boxes) and CSGs (blank box filled with black spots), and the right-*Y*-axis represents the percentage of BSGs (red fold line) and CSGs (green fold line) in each types of intron number.

The genic features of BSGs, CSGs, and ECGs were characterized to determine whether significant differences exist between orphan genes and ECGs (Table 1). On average, the gene and protein lengths of BSGs and CSGs were significantly shorter than those of ECGs (one-way analysis of variance (ANOVA); *p* < 0.001). Similarly, the gene and protein lengths of orphan genes were significantly shorter than evolutionary-conserved genes in rice, *Arabidopsis*, and sweet orange [7,9,11]. However, the average intron length of BSGs and CSGs were significantly longer than that of the ECGs (one-way ANOVA; *p* < 0.001). The average exon number per BSG and CSG gene was significantly smaller than that of the ECGs (one-way ANOVA; *p* < 0.001), and this phenomenon was similar to the findings in sweet orange, rice, and *Arabidopsis* [7,9,11]. Nevertheless, the coding sequence (CDS) and gene GC (guanine cytosine) contents of both BSGs and CSGs were significantly higher than that of ECGs (one-way ANOVA; *p* < 0.01), and this was consistent with the results of former studies on rice, *Arabidopsis*, and *Populus trichocarpa* [7,11,35]. Intron GC content of both BSGs and CSGs was also significantly higher than that of the ECGs (one-way ANOVA; *p* < 0.01), however, studies showed that orphan genes in sweet orange, *P. trichocarpa*, and *Drosophila* possessed lower intron GC content [11,35,37]. Additionally, to study the intron–exon structure of BSGs and CSGs, we examined intron composition by dividing gene structures into seven types: intron-less, one intron, two introns, three introns, four introns, five introns, and greater than or equal to six introns per gene in both sets (Figure 3). A total of 826 (53.64%) BSGs and 759 (41.61%) CSGs were intron-less genes, and BSGs and CSGs contained 24.81% and 27.25% one intron genes, respectively. Moreover, other types of intron number accounted for less than 15.00%. Genic feature analyses showed that BSGs and CSGs were distinct gene sets compared with ECGs.

**Figure 4:**
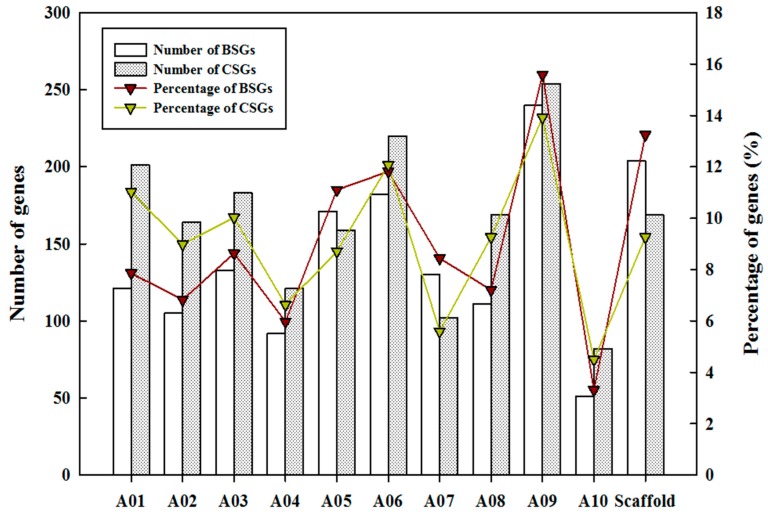
The numbers of BSGs and CSGs distributed on each chromosome of *B. rapa*. Both numbers and percentages are shown. The *X*-axis represents ten chromosomes and scaffold of *B. rapa*, and the left-*Y*-axis indicates the number of BSGs (blank boxes) and CSGs (blank box filled with black spots) on each chromosome, while the right-*Y*-axis represents the percentage of BSGs (red fold line) and CSGs (green fold line) on each chromosome.

Moreover, the physical maps of BSGs and CSGs across the 10 *B. rapa* chromosomes were drawn according to the information from the *B. rapa* genome (Figures S1 and S2). The physical maps showed relatively even distributions on certain chromosomes, and this was consistent with the conclusions obtained in other species, such as sweet orange [11]. Each orphan gene set had uniform distribution on each chromosome within the different *B. rapa* chromosomes (Figure 4), and the highest percentage of BSGs and CSGs gene numbers were distributed on chromosome A09, accounting for 15.58% and 13.93%, respectively. Spearman’s test was performed to determine if the percentage of BSGs and CSGs on each chromosome correlated with the length of the *B. rapa* chromosomes, and the results indicated that the number of BSGs (*p* = 0.00001, *r* = 0.945) on each chromosome correlated with the length of their respective chromosome. Furthermore, CSGs (*p* = 0.025, *r* = 0.697) were correlated with the length of their respective chromosomes when number of CSGs located at the scaffold were removed.

### 2.3. Verification of the BSGs in B. rapa and Other Species

Before verification, 1540 BSGs were further classified to identify different types of BSGs, which were searched against 4 proteomes (*B. nigra*, B genome; *B. oleracea*, C genome; *B. juncea*, AB genomes; *B. napus*, AC genomes) and PUTs within *Brassica* excluding *B. rapa* via BLAST programs, and other procedures to identify different types of BSGs similar to the identification of CSGs in Figure 2. The results showed that 1540 BSGs were divided into five types, (1) real A subgenome-specific, BSGs specific to A subgenome and genes only can be found in *B. rapa* (Table 2); (2) A subgenome-specific, genes appeared in *B. rapa* and the A subgenome that originated from AB and AC genomes; (3) A/B subgenome-specific, genes cannot be found in the C subgenome, and some genes also occurred in AC genomes because they contained A subgenome; (4) A/C subgenome-specific, homologs can be found in A and C subgenomes and the AC genomes, but not the B subgenome, and homologs also occurred in AB genomes because they contain A subgenome; and (5) A/B/C subgenome-specific, homologs can be found in all three subgenomes. Taken together, real A subgenome-specific, A, A/B, A/C, and A/B/C subgenome-specific types contained 529, 474, 130, 229 and 178 BSGs, respectively.

Then, 145 out of 529 real A subgenome-specific BSGs were selected and verified via a total of 51 homozygous self-inbred plant materials from *Brassica* and Cruciferae species (Tables S1 and S2). The 145 BSGs were first verified in *A. thaliana*, and target bands were not amplified. The verification was then conducted in Chiifu, and 52 BSGs were successfully amplified (Figure S3). The 52 BSGs were subsequently validated in 49 plant materials from six *Brassica* genomes. According to the verification results (Table S2), modules for different BSG types identified by PCR from 49 *Brassica* species are listed in Figure S4. The results showed that the real A subgenome-specific type only contained one gene (e.g., *BraA10000785*), which was only amplified in *B. rapa*, and 15 BSGs (10.34%) belonging to A subgenome-specific type (e.g., *BraA10001918*) (Figure S4). A/B and A/C subgenome-specific types account for 8.97% (13 genes) and 3.45% (five genes), respectively, and the remaining 18 BSGs (12.41%) were classified into A/B/C subgenome-specific types. Some genes identified in A/B (e.g., *BraA10000178*), A/C (e.g., *BraSca000608*), and A/B/C (e.g., *BraA10000717*) subgenome-specific types also occurred in BC genomes because they contain B and C subgenomes.

### 2.4. Expression Patterns of BSGs during Different B. rapa Developmental Stages

**Figure 5:**
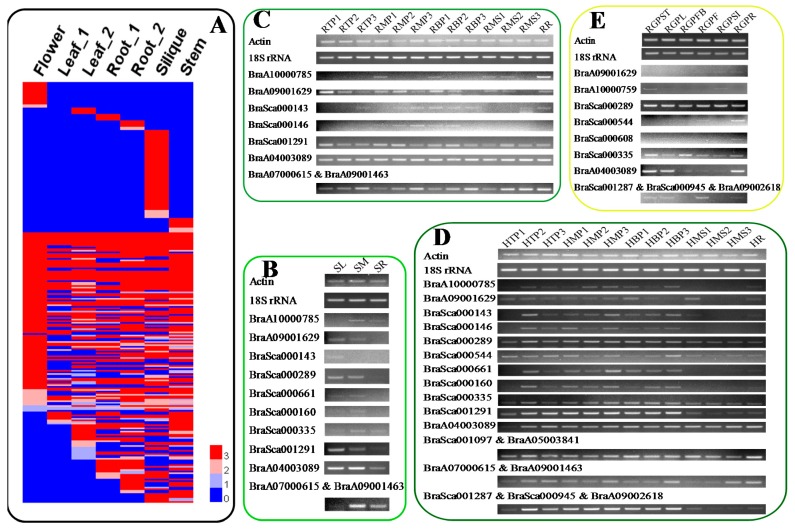
Expression patterns of BSGs during different *B. rapa* developmental stages. (**A**) Heat map of BSGs in different tissues of *B. rapa* (fragments per kilo bases of exons for per million mapped reads—FPKM > 2). Values in the figure are FPKM changes of different tissues. The column represents different tissues (Root, stem and leaf tissues were obtained from seven-week old plants, and two samples of root and leaf tissues were collected from different batches of plants; flower tissue was obtained from blooming plants and silique tissue was generated from 15-day old plants after pollination. Described in Tong, et al., 2013), and row represents individual genes. Gene expression patterns were analyzed by semi-qPCR. *B. rapa* 18S rRNA and Actin genes were used as the internal control. (**B**) Seedling stage. (**C**) Rosette stage. (**D**) Heading stage. (**E**) Reproductive stage. Other details were listed in materials and methods. “&” indicated the gene belonging to multiple copy genes.

Although the potential functions of BSGs are unknown, the expression pattern of a gene is often correlated with its function [11]. Therefore, BSGs expression patterns were analyzed. The protein sequences of 1540 BSGs were queried against *B. rapa* EST sequences and PUTs via TBLASTN (*E*-value cutoff of 1 × 10^−3^; query cover and identity ≥70%), but only 73 unique genes with expression evidence were obtained (56 BSGs in Table S3 and 65 BSGs in Table S4). Besides, only four out of 52 BSGs validated in this study have expression evidence support. EST sequences and PUTs may not be comprehensive enough, so raw RNA-Sequence (Seq) data from seven *B. rapa* (Chiifu) tissues [33] were reassembled under the *B. rapa* genome (v 2.5). These data were then used to further analyze the expression patterns of BSGs. A total of 753 BSGs (263 unique genes) were supported by RNA-Seq data (fragments per kilo bases of exons for per million mapped reads—FPKM > 2 for least one tissue [11]) (Figure 5A, Table S5), and 105 BSGs with high expression abundance (genes with FPKM > 50 in at least one tissue [33]) that contained 42 unique genes. Thirty-three genes showed constitutive expression in all tissues (FPKM > 2). BSGs to the amounts of 16, 0, 4, 4, 6, 55, and 9 were specifically expressed in seven different tissues, respectively (FPKM > 2) (Table S5). Thus, we assumed that BSGs may play vital roles in other developmental stages or different tissues.

We then analyzed the expression patterns of BSGs using semi-qPCR. Samples from different tissues of *B. rapa* plants during heading (seedling stage, rosette stage, and heading stage) and reproductive stage were used, and 52 successfully amplified BSGs (Figure S3) were analyzed using semi-qPCR. Interestingly, 20 (38.46%) out of the 52 BSGs were expressed during more than one developmental stage (Figure 5B–E), however, only eight out of these 52 BSGs were supported by RNA-Seq data (FPKM > 2 for least one tissue) (Figure 5A, Table S5). Two BSGs (*BraA04003089* and *BraA09001629*) exhibited constitutive expression at all stages (FPKM > 2). Some genes showed tissue-specific expression, including *BraSca000146* (specifically expressed in the middle and bottom points of rosette stage leaves; Figure 5C), or organ-specific expression, including *BraSca000143* (specifically expressed in leaves at the seedling stage; Figure 5B). Furthermore, 32 BSGs (61.54%) were not detected in any of the four developmental stages, and we speculated that these BSGs could be induced by biotic or abiotic stresses.

### 2.5. Expression Patterns of BSGs in B. rapa after P. brassicae Infection

**Figure 6:**
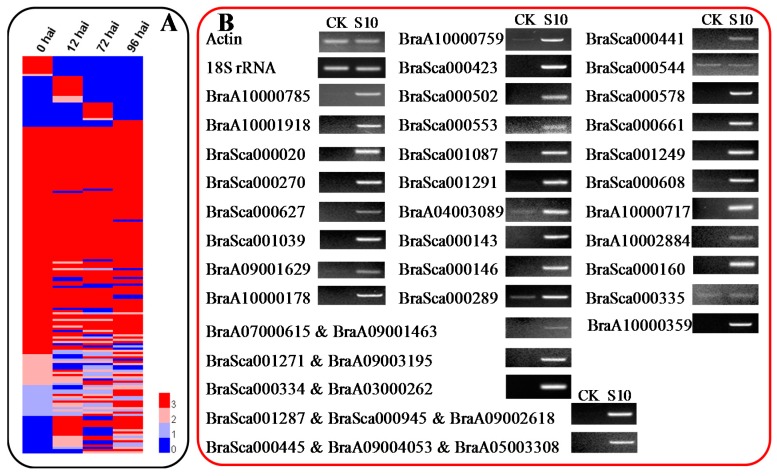
Expression patterns of BSGs in *B. rapa* after *P. brassicae* infection. (**A**) Heat map of BSGs after *P. brassicae* treatment of Chinese cabbage (FPKM > 2). Zero, 12, 72, and 96 hai (hai: hours after inoculation) represents clubroot-susceptible Chinese cabbage (BJN3-2) that was infected by Pb. Gene expressions were analyzed by semi-qPCR. *B. rapa* 18S rRNA and Actin genes were used as the internal control. (**B**) Pb treatment. Other details were listed in materials and methods. “&” indicated the gene belonging to multiple copy genes.

Studies showed that orphan genes are often associated with biotic and abiotic stress, and they could function in stress-response signal transduction pathways [5,15]. Previously, using RNA sequencing, transcriptome profiling was compared between clubroot-resistant and clubroot-susceptible *B. rapa* genotypes after infection with Pb during early infection [32]. We reassembled respective RNA sequencing data to analyze the BSGs expression patterns, and the results indicated that 180 unique BSGs were supported by RNA-Seq data (FPKM > 2 for least one sampling stage [11]) (Figure 6A, Table S6). Furthermore, 24 BSGs exhibited high expression abundance for least in one sampling stage (FPKM > 50 [33]). Seventy-eight BSGs displayed constitutive expression at every point (FPKM > 2), and 32 BSGs showed period-specific expression (FPKM > 2) (Table S6). Previous research indicated that *P. brassicae* was blocked at middle or late stages of infection in resistant *Brassica* crops [38]. We assumed that BSGs may play more important roles in middle or late periods (4–20 days after inoculation (dai)). To confirm this hypothesis, roots from *B. rapa* plants were collected at 10 dai with Pb (referred to as CK and S10), and 52 successfully amplified BSGs in Chiifu were analyzed using semi-qPCR. As expected, 78.85% (41 genes) of analyzed genes responded to Pb treatments, and 39 out of 41 BSGs were up-regulated (Figure 6B). However, only 8 out of these 52 BSGs have transcript support (FPKM > 2) (Figure 6A, Table S6). Thus, our results strongly suggesting that BSGs play important roles in Pb stress responses.

### 2.6. Heading Stage-Specific Expression Patterns of BSGs1 in Chinese Cabbage

**Figure 7:**
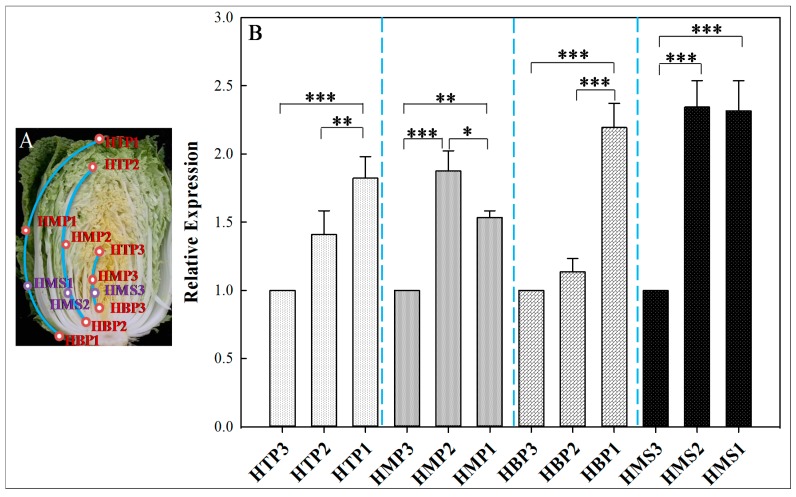
Heading stage-specific expression patterns of *BSGs1* in Chinese cabbage. (**A**) Heading stage sampling module. (**B**) Expression patterns of *BSGs1* within heading stage. Data were normalized to *B. rapa* 18S rRNA and Actin expression level and presented as mean ± SE of three independent measurements; * *p* ≤ 0.05, ** *p* ≤ 0.01, *** *p* ≤ 0.001; *p* > 0.05 for those with no marks.

Studies indicated that orphan genes are often associated with species-specific traits [5]. Heading traits are important, specific traits that are of significant economic importance to Chinese cabbage of *Brassica* A subgenome and the *Brassica* C subgenome [39,40,41]. We assumed that A/C subgenome-specific BSGs play vital roles in leafy head formation of Chinese cabbage. Five BSGs belonging to the A/C subgenome-specific type were revealed by PCR results in this study (Table S2), and only *BraA05003841* and *BraSca001097* were specifically expressed during the heading stage (Figure 5D, Table 3). Further analysis showed that these two BSGs share common CDS and protein sequences, and the only difference was the chromosomal locations. Thus, we considered these two genes as one single gene named, *BSGs1*. The expression patterns of *BSGs1* were analyzed using qRT-PCR to investigate the potential role of *BSGs1* in the heading stage. Three parts (top, middle, and bottom) of outer leaf (HTP1, HMP1, and HBP1), middle leaf (HTP2, HMP2, and HBP2), and inner leaf (HTP3, HMP3, and HBP3) were collected, and the middle point of the middle ribs of outer, middle, and inner leaves (HMS1, HMS2, and HMS3) were collected from the heading stage of the Chinese cabbage (Figure 7A). The expression of *BSGs1* was significantly accumulated in both outer and middle leaves (Figure 7B). At the top and bottom parts, *BSGs1* expression increased significantly from inner to outer leaves. Regarding the middle part, the expression peak can be observed at HMP2. For the middle rib, *BSGs1* showed higher expression levels at HMS1 and HMS2 than at HMS3. Taken altogether, *BSGs1* may provide possible indications of exploring the potential functions of orphan genes during leafy head formation of Chinese cabbage.

## 3. Discussion

### 3.1. Identification of Orphan Genes in B. rapa

Using increasingly stringent BLAST searching criteria, we identified 1540 BSGs, 1824 CSGs, and 45,462 ECGs based on the genome of *B. rapa* (Figure 1 and Figure 2). The analysis included comparisons with 89 complete genomes released from Phytozome 12.1 and BRAD; 267 plant species PUTs from PlantGDB; and comparisons with UniProt-KB from UniProt, Nrdb, other EST databases, and the Nucleotide collection (Nr/Nt) from NCBI. The continuous updating of plant genomes and various databases lowered false positives associated with the analysis of orphan genes. A total of 1324 lineage-specific genes in *A. thaliana* (ALSGs) [9] and 165 *Arabidopsis* species-specific genes (ASS) [10] were identified under TAIR8 and TAIR7 releases (two *A. thaliana* genome versions), respectively. This phenomenon could be explained by the fact that a higher genome version was used, leading to a significantly higher accuracy of identification of orphan genes. Thus, we adopted a higher genome version of *B. rapa* (v 2.5) that contains 48,826 gene modules. While some false positives are possible, our method, based on limited but available genome databases, led to the identification of BSGs and CSGs, constituting an important step towards the identification of novel *B. rapa* genes.

The number of BSGs (1540) in *B. rapa* is slightly higher than that detected in sweet orange (1039 orphan genes) [11] and *Arabidopsis* (1324 *Arabidopsis* lineage-specific genes) [9], and this is mainly because of genome size differences. Another study identified 165 *Arabidopsis* species-specific genes using a BLAST *E*-value cut-off of 1 × 10^−1^ [10], and this result was much lower than the number of BSGs identified in this study. The relaxed *E*-value cut-off in the earlier study likely caused a higher false negative rate, so some true orphan genes were likely missed. The various databases that were used in this study were much richer than those used in the former study. We identified more CSGs (3.74%) than BSGs (3.15%), which was in accordance with the evolutionary histories of the *B. rapa* genome [21]. As well as β and α whole-genome duplications (WGD) are shared by most Cruciferae species, and the recent *Brassica*-specific whole-genome triplication (WGT) has greatly influenced extant *Brassica* genome diversification [42]. Thus, we hypothesized that the majority of the ECGs might have evolved before the ancient α WGD. Furthermore, after α WGD and Cruciferae-specific WGT, *Brassica* species likely evolved to respond to specific traits or adaptation mechanisms associated with each lineage under artificial selection during domestication and breeding.

### 3.2. Characterizing BSGs and CSGs

BSGs and CSGs had shorter genes than ECGs as a result of fewer numbers of exons per gene (Table 1) and a relatively lower percentage of multi-exon genes (Figure 3). These results were similar to those reported in *A. thaliana* [9], sweet orange [11], yeast [43], and metazoans [20,44]. The GC content of CDS and genes of both BSGs and CSGs increased when compared with that of ECGs (Table 1), which was consistent with findings of lineage-specific genes in rice, *Arabidopsis*, and *Populus trichocarpa* [7,11,35]; however, that higher intron GC content of BSGs and CSGs when compared with ECGs were in contrast with the findings in sweet orange, *P. trichocarpa*, and *Drosophila* [11,35,37]. Furthermore, 53.64% of BSGs and 41.61% of CSGs were intron-less genes (Figure 3), and this was much higher than that of ECGs (22.86%). On one hand, the higher proportion of intron-less genes within BSGs and CSGs is likely the result of recent lineage-specific expansion, which might have generated new genes through retro-transposition [11]. On the other hand, intron-less genes might be associated with some lineage-specific and species-specific characteristics during the process of species evolution, and intron-less genes are likely the main reasons for the existence of biodiversity [45]. Studies showed that gene origin and extinction occurred under a vast, dynamic reservoir [5], and there is a balance between gene emergence and gene loss [46]. Some orphan genes will be lost, and others may become fixed before forming a new gene family by increasing the number of introns [46]; therefore, further studies are needed to test this phenomenon. Although the chromosome distributions of 13.25% BSGs and 9.27% CSGs are still unknown, the expression results of semi-qPCR analyses indicated that only one out of 32 BSGs located on a scaffold was not detected (Table 3 and Table S2). We then hypothesized that these genes were not artifacts of *B. rapa* genome annotation, but rather real genes. These findings are similar to those reported in sweet orange [11]. Overall, BSGs and CSGs in *B. rapa* are associated with more radical evolution when compared with ECGs.

### 3.3. Verification of BSGs

1540 BSGs were further classified into five types via BLAST programs, a higher proportion of real A subgenome-specific type (34.35%) and a lower proportion of A/B/C subgenome-specific type (11.56%) were obtained (Table 2), we assumed that the formation of some specific characteristics of *B. rapa* may need to evolve more specific genes. Meanwhile, the impact of incompleteness of genome sequencing of *B. rapa* cannot be ruled out. In order to confirm real A subgenome-specific BSGs, 145 out of 529 BSGs were selected, PCR assays were performed based on the validation of BSGs within 51 homozygous self-inbred plant materials, and no validation experiments were previously reported. The results showed that 93 out of 145 BSGs were not detected in the *B. rapa* reference genome, as well as other *Brassica* materials and *A. thaliana* (Table S2), and these BSGs have no expression evidence support by ESTs, PUTs, or RNA-Seq data. Thus, we hypothesized that the inexistence of these 93 BSGs were mainly caused by sequence assembly errors. Regarding the remaining 52 BSGs within non-*Brassica* species, no target bands were amplified in *A. thaliana*. Subgenome-specific BSGs (such as *BraA10001918* and *BraSca000020*) can not only be amplified in *B. rapa* species, but also in *B. juncea* and *B. napus* (Figure S4 and Table S2). Then, the sequencing verification of target bands amplified from *B. juncea* and *B. napus* suggested that they possess higher similarity to *B. rapa* sequences (identity and query cover greater than 90%, data not shown). Therefore, we proposed that these target bands likely originate in the A subgenome of AB and AC genomes. A higher percentage of A/B/C subgenome-specific BSGs and a lower proportion of real A subgenome-specific BSGs (such as *BraA10000785*) were verified, mainly as a result of A subgenome from *B. rapa*, *B. napus*, and *B. juncea* showing strong co-linearity [41,47]. Although the more stringent BLAST searching criteria were used in this study, the analysis of genome sequencing of *B. juncea*, *B. nigra* [47], *B. napus* [48], and *B. oleracea* [41] indicated that few genome assemblies are still missing (also another possible reason). The evolutionary time from the *A. thaliana* genome to the *B. rapa* genome was far longer than the evolutionary time from *B. rapa* to *B. juncea*, *B. napus,* and *B. carinata*, and this may result in a higher percentage of common genes between *B. rapa* and other *Brassica* genomes [42]. The genome of *A. thaliana* was diverged at 17–18 million years ago (Mya) before Cruciferae-specific WGT [49], *B. rapa*, and *B. oleracea* genome divergence occurred at about 4.6 Mya, while *B. nigra* genome diverged 1.6 Mya earlier than *B. rapa* and *B. oleracea* [50]. Nevertheless, genome divergence of three amphidiploid species (AB, AC, and BC genomes) occurred at 0.075 Mya, leading to the hybridization of three diploid species (A, B, and C genome) [48], which indicated that most genes in *B. rapa* may also exist in other *Brassica* species. Thus, real A subgenome-specific BSGs (such as *BraA10000785*) in the *B. rapa* genome still need to be validated in future, and whether real A subgenome-specific BSGs are specific to Chinese cabbage still need to be determined.

### 3.4. Expression Analysis of BSGs at Different Developmental Stages and Response to P. brassicae

The potential functions of orphan genes cannot be assigned by cause if hits were found in any other plant genomes or various databases. Gene expression patterns are often correlated with functions, and expression analyses are effective when determining the potential functions of orphan genes [11]. The protein sequences of 1540 BSGs were used to compare *B. rapa* ESTs and PUTs in NCBI and PlantGDB via TBLASTN. However, only 73 unique genes had expression support, and this might have resulted from the incompleteness of EST sequences and PUTs. Thus, the raw RNA-Seq data of seven *B. rapa* (Chiifu) tissues [33] was reassembled, and the results showed that only 263 unique BSGs were supported by RNA-Seq data (Figure 5A and Table S5). Semi-qPCR was also carried out using the 52 validated BSGs to confirm the expression patterns of BSGs in different tissues. Only 38.46% of the 52 BSGs exhibited expression during more than one developmental stage (Figure 5B–E). The numbers of expressed BSGs were much lower, and this is likely because BSGs may be expressed under limited conditions or when expression levels are too low to be detected by traditional transcript profiling [9].

Studies showed that *P. brassicae* is a soil-borne, obligate, and biotrophic pathogen that attacks *Brassica* crops, leading to clubroot and subsequent reductions in crop yield [32]. Thus, the raw RNA-Seq data from four points (0, 12, 72, and 96 hai) of clubroot-susceptible Chinese cabbage (BJN3-2) infected by *P. brassicae* [32] was reassembled. The results showed that only 11.69% unique BSGs were supported by RNA-Seq data (FPKM > 2) (Table S6). However, the results of semi-qPCR analysis showed 78.85% BSGs response to Pb treatment at 10 dai (Figure 6B). These results confirmed that BSGs may play vital roles in Pb stress responses.

Based on the results of semi-qPCR, 43 (82.69%) out of 52 BSGs identified in this study have expression evidence in one or more developmental stage and Pb treatments (Table 3). However, only 9 (17.31%) out of 52 BSGs have expression evidence support from RNA-Seq data. Likewise, almost 65% of lineage-specific genes in *P. trichocarpa* (PtSS) exhibited no expression evidence when compared with EST databases using TBLASTN. However, subsequent qRT-PCR assays indicated that only 34.62% of PtSS with no expression evidence showed detectable expression [35]. Two BSGs were found continuously expressed at all analyzed developmental stages or treatments by semi-qPCR, which suggested that some BSGs may act as housekeeping genes and play roles in basal cellular function maintenance in *B. rapa* (Figure 5 and Figure 6, and Table 3), which is similar to the results of previous studies in sweet orange [11]. Approximately 34.62% out of 52 BSGs identified in this study presented expression during two or more developmental stages, and some BSGs displayed tissue-specific (such as *BraSca000146*) or organ-specific expression (such as *BraSca000143*) (Table 3, Figure 5 and Figure 6). However, most BSGs have lower expression levels. Likewise, the expression patterns of LSGs in *A. thaliana* also indicated a greater extent of tissue specificity and lower expression levels [3].

According to the semi-qPCR results, 23 BSGs were specifically expressed under Pb treatment, indicating that BSGs may be important for responses to Pb treatment and the defense system. Thus, the role of BSGs in *B. rapa*–*P. brassicae* interactions requires intensive study in future, which may provide new approaches to facilitate resistance breeding of Chinese cabbage and Cruciferae crops. Furthermore, previous studies showed that high stress-specificity has been highlighted as a characteristic related to orphan genes [3,51,52], which was consistent with our findings. However, the expression profiles for nine BSGs were still not detected at any developmental stage or Pb treatment (Table 3). We hypothesized that some BSGs are likely to be expressed in limited tissues, organs, stages, or stress treatments that were not determined. Similar results also occurred in previous studies [11,35]. Thus, the expression patterns of these BSGs should be comprehensively investigated in further studies.

### 3.5. BSGs1 May Be Associated with Leafy Head Formation in Chinese Cabbage

We identified *BSGs1*, which belongs to the A/C subgenome-specific type, which specifically expressed during the heading stage (Figure 5D and Table 3). Studies showed that *B. rapa* (A genome) and *B. oleracea* (C genome) shared typical leaf-heading traits through convergent, subgenome-level parallel selection of paralogous genes [26]. The heading stage-specific expression patterns of *BSGs1* in Chinese cabbage were observed using qRT-PCR (Figure 7B). *BSGs1* expression significantly increased from inner to outer leaves at the top and bottom points of leaves, and the expression levels at HMS2 and HMS1 were significantly accumulated compared with HMS3. These findings suggested that *BSGs1* may be associated with the formation of a leafy head of Chinese cabbage, and further functional experiments (such as over-expression and gene silencing) are essential for the verification of functional *BSGs1* mechanisms and the process of leafy head formation in Chinese cabbage, which may provide insight into exploration of the potential roles of A/C subgenome-specific BSGs during the leafy head formation of Chinese cabbage.

## 4. Materials and Methods

### 4.1. Genome Datasets

The proteome sequence of *B. rapa* (version 2.5) was obtained from the *Brassica* database (BRAD, http://brassicadb.org/brad/index.php). To identify ECGs, CSGs, and BSGs, 93 complete genomes released in Phytozome 12.1 and BRAD were used (76 non-Cruciferae proteomes, 13 Cruciferae genomes excluding *Brsssica* species, and 4 proteomes within *Brassica* excluding *B. rapa*). All genomes were downloaded from Phytozome 12.1 (https://phytozome.jgi.doe.gov/pz/portal.html) and BRAD in December 2017. Plant genome database (PlantGDB) generated unique transcripts (PUTs) from 267 plant species (259 non-Cruciferae species PUTs and 8 non-*Brassica* species PUTs within Cruciferae) were downloaded from PlantGDB (http://www.plantgdb.org/prj/ESTCluster/progress.php) in December 2017. UniProt-KB (Release 2017_12) was downloaded from UniProt (ftp://ftp.ebi.ac.uk/pub/databases/uniprot/knowledgebase/). Non-redundant database (Nrdb), other ESTs databases, and nucleotide collection (Nr/Nt) analyses were performed in NCBI (https://www.ncbi.nlm.nih.gov/) in December 2017.

### 4.2. Homolog Search

Three sets of genes within *B. rapa* were identified based on a homolog search using BLASTP, TBLASTN, and BLASTN with an *E*-value cutoff of 1 × 10^−3^ [3], and BLAST searches were performed between 12 December 2017 and 12 January 2018 (Figure 1 and Figure 2). *B. rapa* genes were classified into three sets: BSGs, CSGs, and ECGs. Here, BSGs included genes for which we could not find any homologs in non-*Brassica* species, and CSGs included genes for which we could find at least one homolog in Cruciferae species only, excluding *Brassica* species. ECGs were genes with at least one homolog outside of Cruciferae. When compared with sequences in other EST databases and the Nr/Nt database in NCBI using TBLASTN and BLASTN, manual inspection of the resulting alignments was essential (*E*-value < 1 × 10^−3^, query cover and identity <70%).

### 4.3. Genic Features and Physical Maps

Whole genome *B. rapa* information was downloaded from BRAD to observe the characteristics of the three *B. rapa* gene sets. Perl scripts were used to calculate the number of exons and introns; CDS length; gene length; protein length; intron length; and GC content of CDS, genes, and introns. One-way analysis of variance (ANOVAs) tests were used to identify significant differences between different sets of orphan genes and ECGs. Chromosome localization information was extracted from chromosomal sequences, and Spearman’s test was performed using SPSS 19.0 [53] to determine whether the percentage of BSGs and CSGs in each chromosome were correlated with chromosome length. Physical maps of BSGs and CSGs were constructed using the chromosomal position of each gene as a starting point along the chromosomes using MapChart 2.32 [54].

### 4.4. Verification of BSGs

The reference genome sequence of the *B. rapa* Chiifu cultivar and 50 other homozygous self-inbred plant materials from *B. rapa* (eight species), *B. nigra* (nine species), *B. oleracea* (eight species), *B. juncea* (seven species), *B. napus* (six species), *B. carinata* (11 species), and wild-type *A. thaliana* (ecotype Columbia, Col-0) were used in this study (all species identities (ID) are listed in Table S1). All plants were germinated in soil in 18 or 20 cm pots in a greenhouse, and plants were maintained at a 16-h photoperiod at about 20 °C at 60–70% humidity. Young leaf tissues from nine individuals (three biological replicates with three plants per replicate) were collected after two weeks of growth and maintained at −80 °C for DNA isolation. DNA was isolated from leaves using the cetyl trimethyl ammonium bromide (CTAB) method with slight alterations [55]. Afterwards, samples were treated with DNase-free RNaseA to remove RNA contamination, and the DNA concentration was determined using a Nanodrop spectrophotometer (Thermo Fisher Scientific, Waltham, MA, USA) and 1% agarose gel electrophoresis. DNA was then diluted to 20 ng·μL^−1^ for PCR reactions, which were conducted on a Veriti Thermal Cycler (Thermo Fisher Scientific, Waltham, MA, USA). PCR amplification conditions were based on the length of the target fragment, and 1% agarose gel electrophoresis was used to visualize PCR products. Primer Premier 5.0 software was used to designed primer pairs, and primer pairs used for the validation of BSGs are listed in Table S7.

### 4.5. Expression Evidence and RNA-Sequence (Seq) Data Reassemble

All *B. rapa* PUTs and EST sequences were downloaded from PlantGDB and NCBI EST databases (https://www.ncbi.nlm.nih.gov/nucest/) on 13 December 2017, respectively. TBLASTN was used to compared BSGs protein sequences to PUTs and EST sequences, and BSGs expression data from PUTs or EST sequences were determined based on a minimum of 70% identity and at least 70% query cover with an *E*-value cut-off less than 1 × 10^−3^. Raw RNA-Seq data from seven *B. rapa* tissues were downloaded from the sequence read archive (SRA) in NCBI (https://www.ncbi.nlm.nih.gov/sra?term=SRP017757). Raw RNA-Seq data from four points (0, 12, 72, and 96 h after inoculation (hai)) of clubroot-susceptible Chinese cabbage (BJN3-2) that was infected by *P. brassicae* were downloaded from SRA (https://www.ncbi.nlm.nih.gov/sra?term=SRP064840). We mapped sequencing reads to the reference database for the *B. rapa* genome (v 2.5, http://brassicadb.org/brad/) using TopHat [56]. Before mapping, the first 15 bp of reads were trimmed using Fastx Toolkit (v 0.013) [57], and the fragments per kilo bases of exons for per million mapped reads (FPKM) expression values of transcripts were calculated using Cufflinks (v 1.3.0) [58]. The standards applied to filter out genes with preferential expression profiles for each tissue or each treatment period were the following. (1) Expression abundance: FPKM > 2 in at least one tissue or period [11]; (2) high expression abundance: FPKM > 50 for at least one tissue [33]; and (3) tissue or period-specific expression where gene expression only appeared in one tissue or period with FPKM > 2.

### 4.6. Different Developmental Stage Sampling

Roots, leaves, and middle ribs were obtained from nine Chiifu individuals (three biological replicates with three plants per replicate) after three weeks of growth, which was defined based on seedling stage (referred to SR, SL, and SM, respectively). Leaves and middle ribs were sampled based on the composition of the oldest and youngest tissues, respectively. Rosette stage and heading stages were sampled until the sixth and eighth weeks, respectively. During the rosette stage, three different leaves (outer leaf, first leaf; middle leaf, 10th leaf; inner leaf, 20th leaf) from nine Chiifu individuals (three biological replicates with three plants per replicate) were sampled. The oldest leaf was number one, and samples were taken from three different positions on three different leaves: (1) the top points of three leaves; (2) the right and left sides from the middle rib; and (3) 2–3 cm (width) of leaf tissue referred to as RTP1-3 from outer to inner leaves. The sampling of the middle (RMP1-3) and bottom (RBP1-3) points of leaves were the same as the top points of the leaves, and the middle points of middle ribs were sampled from three leaves, which were referred to as RMS1-3. During heading stage, three different leaves (outer leaf, first leaf; middle leaf, 20th leaf; inner leaf, 40th leaf) from nine Chiifu individuals (three biological replicates with three plants per replicate) were obtained, and the sampling details were the same as those of the rosette stage (referred to as HTP1-3, HMP1-3, HBP1-3, and HMS1-3). For the reproductive stage, the stems, leaves, flower buds, flowers, siliques, and roots of nine Chiifu individuals (three biological replicates with three plants per replicate) were sampled, and were referred to as RGPST, RGPL, RGPFB, RGPF, RGPSI, and RGPR, respectively. All samples were immediately frozen in liquid nitrogen and stored at −80 °C prior to RNA isolation.

### 4.7. *P. brassicae* Treatment Sampling

Clubroot-susceptible Chinese cabbage (BJN3-2) was inoculated with *P. brassicae* (Pb) suspension. The details of inoculation of Pb were described in a previous study [32]. The inoculated plants were kept at 25 °C under a 16-h photoperiod in a culture room, and the soil was kept moist during the treatment period. Roots from 30 individuals (three biological replicates with 10 plants per replicate) were collected after 10 days’ inoculation (referred to as S10), and roots from 30 individuals (three biological replicates with 10 plants per replicate) without Pb treatment were simultaneously obtained (referred to as the control group (CK)). All samples were immediately frozen in liquid nitrogen and stored at −80 °C prior to RNA isolation.

### 4.8. Total RNA Isolation and cDNA Synthesis

Total RNA was isolated using TRIzol^®^ Reagent (Invitrogen, Carlsbad, CA, USA) based on the manufacturer’s instructions. Samples were then treated with RNase-free DNase to remove genomic DNA contamination, and a Nanodrop spectrophotometer (Thermo Fisher Scientific, Waltham, MA, USA) and 1% formaldehyde gel electrophoresis were used to determine RNA concentrations. RNA was reverse-transcribed to synthesize first-strand cDNA using a PrimeScript™ II 1st Strand cDNA Synthesis Kit (TaKaRa, Kyoto, Japan) according to the manufacturer’s instructions.

### 4.9. Semi-qPCR and qRT-PCR

Primer pairs were designed to amplify specific BSGs using Primer Premier 5.0 software. Semi-qPCR and qRT-PCR primer pairs are listed in Table S7. Semi-qPCR was performed using a Veriti Thermal Cycler (Thermo Fisher Scientific, Waltham, MA, USA) under the following conditions: 5 min at 95 °C followed by 30 cycles at 95 °C for 30 s, 60 °C for 30 s, and 72 °C for 30 s. All the samples of semi-qPCR followed the same reaction volume (20 μL), same cDNA concentration (first-strand cDNA was diluted 10-fold, and 1 μL of the diluted cDNA was used in each 20 μL PCR mixture), and same volume loaded into gel (10 μL). Reaction products were visualized using 2% agarose gel electrophoresis. Previously described methods were used to assess the performance of qPCR reactions [59], and expression level calculations were performed using the 2^−∆∆Ct^ method [53]. Lastly, *B. rapa* 18S rRNA and Actin gene were used as the internal reference genes for both semi-qPCR and qRT-PCR experiments (18SF: GTTCTTAGTTGGTGGAGCGATT, 18SR: ACCTGTTATTGCCTCAAACTTC; ActinF: CGAAACAACTTACAACTCCA, ActinR: CTCTTTGCTCATACGGTCA).

## 5. Conclusions

Our study comprehensively characterized two sets of *B. rapa* orphan genes, BSGs and CSGs, which are specific to *Brassica* and Cruciferae, respectively. Five types of BSGs were obtained via BLAST programs, and 145 out of 529 real A subgenome-specific BSGs were verified using PCR validation from 51 species; the results improved our understanding of *Brassica* genome evolution. Expression analyses of BSGs suggested that some BSGs displayed tissue-, organ-, and stage-specific expression, and the expression of some of these genes can be induced by *P. brassicae*. Moreover, one A/C subgenome-specific BSG (*BSGs1*) was found to be specifically expressed in the heading stage, so, this particular gene may be associated with leafy head formation in Chinese cabbage. This study provides valuable information that may be used to conduct an intensive study on the functions of BSGs in *B. rapa*.

## Figures and Tables

**Table 1:** Genic features of the *Brassica*-specific genes (BSGs), Cruciferae-specific genes (CSGs), and evolutionary-conserved genes (ECGs) of *B. rapa*; orphan genes; and evolutionary-conversed genes of *A. thaliana*, sweet orange, and *P. trichocarpa*.

Feature	BSGs	CSGs	ECGs	ALSGs [9]	*A. thaliana* ECs [9]	Sweet Orange Orphan Genes [11]	Sweet Orange ECs [11]	*P. trichocarpa* Species-Specific Genes [35]	*P. trichocarpa* Conserved Genes [35]
	Mean (SD)	Mean (SD)	Mean (SD)	Mean (SD)	Mean (SD)	Mean (SE)	Mean (SE)	Mean (SD)	Mean (SD)
Exons/gene	1.96 (1.63)	2.29 (1.69)	5.07 (5.06)	1.70 (1.40)	6.00 (5.20)	1.56 (0.87)	4.29 (2.88)	2.15 (1.23)	3.90 (2.46)
CDS length	340.78 (257.43)	473.30 (358.00)	1150.80 (888.33)	—	—	—	—	284.20 (119.20)	252.31 (191.54)
Intron length	309.49 (350.82)	378.21 (547.50)	259.65 (354.95)	227.00 (321.00)	163.00 (172.00)	343.93 (594.69)	362.67 (588.82)	306.87 (392.30)	381.89 (383.70)
Gene length	631.61 (799.75)	907.63 (1016.75)	1991.15 (1815.28)	537.00 (652.00)	2315.00 (1558.00)	705.86 (781.48)	3147.41 (2844.08)	1167.62 (666.80)	2209.07 (1254.57)
Protein length	112.59 (85.81)	156.76 (119.33)	382.60 (296.11)	97.00 (85.00)	431.00 (298.00)	98.58 (70.61)	408.90 (315.22)	—	—
CDS GC (%)	49.58 (5.57)	47.34 (4.81)	46.59 (3.74)	—	—	—	—	46.70 (6.80)	45.00 (3.50)
Intron GC (%)	36.89 (7.97)	36.24 (7.05)	32.43 (5.19)	35.10 (7.60)	32.40 (4.40)	30.99 (7.47)	31.68 (4.92)	22.70 (17.00)	27.70 (13.00)
Gene GC (%)	46.98 (7.35)	44.50 (6.22)	42.29 (5.35)	41.00 (5.10)	39.60 (3.30)	43.00 (8.02)	38.44 (4.36)	42.70 (7.10)	38.60 (3.10)

ALSGs: *A. thaliana* lineage-specific genes; *A. thaliana* ECs: *A. thaliana* evolutionary conserved genes; Sweet orange ECs: sweet orange evolutionary conserved genes. CDS: coding sequence. GC: guanine cytosine. “—” represents the relevant datas are not calculated in the corresponding references.

**Table 2:** Classification results of 1540 BSGs via BLASTP programs in *B. rapa*.

Types	Genomes *	Gene Numbers	Percentage (%)
Real A subgenome-specific	A	529	34.35
A subgenome-specific	A/AB	150	9.74
	A/AC	231	15.00
	A/AB/AC	93	6.04
A/B subgenome-specific	A/B	32	2.08
	A/B/AB	44	2.86
	A/B/AC	16	1.04
	A/B/AB/AC	38	2.47
A/C subgenome-specific	A/C	39	2.53
	A/C/AB	13	0.84
	A/C/AC	113	7.33
	A/C/AB/AC	64	4.16
A/B/C subgenome-specific	A/B/C	4	0.26
	A/B/C/AB	8	0.52
	A/B/C/AC	20	1.30
	A/B/C/AB/AC	146	9.48
	Total	1540	100.00

“*” represents the different genomes in genus *Brassica*, A genome, *B. rapa*; B genome, *B. nigra*; C genome, *B. oleracea*; AB genomes, *B. juncea*; AC genomes, *B. napus*; BC genomes, *B. carinata.*

**Table 3:** Different expression patterns of BSGs within different developmental stages or Pb treatment via semi-qPCR.

Developmental Stages or Treatment	Gene Numbers
Seedling stage/Rosette stage/Heading stage/Reproductive stage/Pb treatment	2
Seedling stage/Rosette stage/Heading stage/Pb treatment	5
Seedling stage/Heading stage/Reproductive stage/Pb treatment	2
Seedling stage/Heading stage/Pb treatment	2
Rosette stage/Heading stage/Pb treatment	1
Heading stage/Reproductive stag/Pb treatment	4
Reproductive stage/Pb treatment	2
Heading stage	2
Pb treatment	23
Expression was not detected	9
Total	52

## Supplementary Materials

The following are available online at http://www.mdpi.com/1422-0067/19/7/2064/s1.

## Abbreviations

BSGs	*Brassica*-specific genes
CSGs	Cruciferae-specific genes
ECGs	evolutionary-conserved genes
LSGs	lineage-specific genes
ORFans	opening reading frames with no detectable sequence similarity to any other ORF in a targeted database
TRPs	tandem repeat proteins
TRGs	taxonomically restricted genes

## References

[1] Amiri H., Davids W., Andersson S.G. (2003). Birth and death of orphan genes in *Rickettsia*. Mol. Biol. Evol..

[2] Beike A.K., Lang D., Zimmer A.D., Wüst F., Trautmann D., Wiedemann G., Beyer P., Decker E.L., Reski R. (2015). Insights from the cold transcriptome of Physcomitrella patens: Global specialization pattern of conserved transcriptional regulators and identification of orphan genes involved in cold acclimation. New Phytol..

[3] Donoghue M.T., Keshavaiah C., Swamidatta S.H., Spillane C. (2011). Evolutionary origins of Brassicaceae specific genes in *Arabidopsis thaliana*. BMC Evol. Biol..

[4] Tautz D., Domazet-Lošo T. (2013). The evolutionary origin of orphan genes. Nat. Rev. Genet..

[5] Arendsee Z.W., Li L., Wurtele E.S. (2014). Coming of age: Orphan genes in plants. Trends Plant Sci..

[6] Dujon B. (1996). The yeast genome project: What did we learn?. Trends Genet..

[7] Campbell M.A., Zhu W., Jiang N., Lin H., Ouyang S., Childs K.L., Haas B.J., Hamilton J.P., Buell C.R. (2007). Identification and characterization of lineage-specific genes within the Poaceae. Plant Physiol..

[8] Guo Y.L. (2013). Gene family evolution in green plants with emphasis on the origination and evolution of *Arabidopsis thaliana* genes. Plant J..

[9] Lin H., Moghe G., Ouyang S., Iezzoni A., Shiu S.H., Gu X., Buell C.R. (2010). Comparative analyses reveal distinct sets of lineage-specific genes within *Arabidopsis thaliana*. BMC Evol. Biol..

[10] Yang X., Jawdy S., Tschaplinski T.J., Tuskan G.A. (2009). Genome-wide identification of lineage-specific genes in *Arabidopsis*, Oryza and Populus. Genomics.

[11] Xu Y., Wu G., Hao B., Chen L., Deng X., Xu Q. (2015). Identification, characterization and expression analysis of lineage-specific genes within sweet orange (*Citrus sinensis*). BMC Genom..

[12] Li L., Foster C.M., Gan Q., Nettleton D., James M.G., Myers A.M., Wurtele E.S. (2009). Identification of the novel protein QQS as a component of the starch metabolic network in *Arabidopsis* leaves. Plant J..

[13] Li L., Wurtele E.S. (2015). The *QQS* orphan gene of Arabidopsis modulates carbon and nitrogen allocation in soybean. Plant Biotechnol. J..

[14] Li L., Zheng W., Zhu Y., Ye H., Tang B., Arendsee Z.W., Jones D., Li R., Ortiz D., Zhao X. (2015). *QQS* orphan gene regulates carbon and nitrogen partitioning across species via NF-YC interactions. Proc. Natl. Acad. Sci. USA.

[15] Perochon A., Jianguang J., Kahla A., Arunachalam C., Scofield S.R., Bowden S., Wallington E., Doohan F.M. (2015). *TaFROG* encodes a Pooideae orphan protein that interacts with *SnRK1* and enhances resistance to the mycotoxigenic Fungus *Fusarium graminearum*. Plant Physiol..

[16] Yadeta K.A., Valkenburg D.-J., Hanemian M., Marco Y., Thomma B.P.H.J. (2014). The Brassicaceae-specific *EWR1* gene provides resistance to vascular wilt pathogens. PLoS ONE.

[17] Luhua S., Hegie A., Suzuki N., Shulaev E., Luo X., Cenariu D., Ma V., Kao S., Lim J., Gunay M.B. (2013). Linking genes of unknown function with abiotic stress responses by high-throughput phenotype screening. Physiol. Plant..

[18] Newman A.M., Cooper J.B. (2011). Global analysis of proline-rich tandem repeat proteins reveals broad phylogenetic diversity in plant secretomes. PLoS ONE.

[19] Shigenobu S., Stern D.L. (2013). Aphids evolved novel secreted proteins for symbiosis with bacterial endosymbiont. Proc. R. Soc. B.

[20] Toll-Riera M., Bosch N., Bellora N., Castelo R., Armengol L., Estivill X., Alba M.M. (2009). Origin of primate orphan genes: A comparative genomics approach. Mol. Biol. Evol..

[21] Kagale S., Robinson S.J., Nixon J., Xiao R., Huebert T., Condie J., Kessler D., Clarke W.E., Edger P.P., Links M.G. (2014). Polyploid evolution of the Brassicaceae during the Cenozoic era. Plant Cell.

[22] Rakow G., Pua E.C., Douglas C.J. (2004). Species Origin and Economic Importance of *Brassica*. Biotechnology in Agriculture and Forestry.

[23] Wang X., Wang H., Wang J., Sun R., Wu J., Liu S., Bai Y., Mun J.-H., Bancroft I., Cheng F. (2011). The genome of the mesopolyploid crop species *Brassica rapa*. Nat. Genet..

[24] Nagaharu U. (1935). Genome analysis in *Brassica* with special reference to the experimental formation of *B. napus* and peculiar mode of fertilization. Jpn. J. Bot..

[25] Cheng F., Mandakova T., Wu J., Xie Q., Lysak M.A., Wang X. (2013). Deciphering the diploid ancestral genome of the Mesohexaploid *Brassica rapa*. Plant Cell.

[26] Cheng F., Sun R., Hou X., Zheng H., Zhang F., Zhang Y., Liu B., Liang J., Zhuang M., Liu Y. (2016). Subgenome parallel selection is associated with morphotype diversification and convergent crop domestication in *Brassica rapa* and *Brassica oleracea*. Nat. Genet..

[27] Zhang C.-W., Wei Y.-P., Xiao D., Gao L.-W., Lyu S.-W., Hou X.-L., Bouuema G. (2016). Transcriptomic and proteomic analyses provide new insights into the regulation mechanism of low-temperature-induced leafy head formation in Chinese cabbage. J. Proteom..

[28] Wang Y., Wu F., Bai J., He Y. (2014). *BrpSPL9* (*Brassica rapa* ssp. *pekinensis* SPL9) controls the earliness of heading time in Chinese cabbage. Plant Biotechnol. J..

[29] Fuchs H., Sacristán M.D. (1996). Identification of a gene in Arabidopsis thaliana controlling resistance to clubroot (*Plasmodiophora brassicae*) and characterization of the resistance response. Mol. Plant-Microbe Interact..

[30] Grsic-Rausch S., Kobelt P., Siemens J.M., Bischoff M., Ludwig-Müller J. (2000). Expression and localization of nitrilase during symptom development of the clubroot disease in *Arabidopsis*. Plant Physiol..

[31] Piao Z.Y., Deng Y.Q., Choi S.R., Park Y.J., Lim Y.P. (2004). SCAR and CAPS mapping of *CRb*, a gene conferring resistance to *Plasmodiophora brassicae* in Chinese cabbage (*Brassica rapa* ssp. *pekinensis*). Theor. Appl. Genet..

[32] Chen J., Pang W., Chen B., Zhang C., Piao Z. (2015). Transcriptome analysis of *Brassica rapa* near-iIsogenic lines carrying clubroot-resistant and -susceptible alleles in response to *Plasmodiophora brassicae* during early infection. Front. Plant Sci..

[33] Tong C., Wang X., Yu J., Wu J., Li W., Huang J., Dong C., Hua W., Liu S. (2013). Comprehensive analysis of RNA-seq data reveals the complexity of the transcriptome in *Brassica rapa*. BMC Genom..

[34] Johnson B.R., Tsutsui N.D. (2011). Taxonomically restricted genes are associated with the evolution of sociality in the honey bee. BMC Genom..

[35] Lin W.-L., Cai B., Cheng Z.-M. (2013). Identification and characterization of lineage-specific genes in *Populus trichocarpa*. Plant Cell Tissue Organ Cult..

[36] Yang L., Zou M., Fu B., He S. (2013). Genome-wide identification, characterization, and expression analysis of lineage-specific genes within zebrafish. BMC Genom..

[37] Domazet-Loso T., Tautz D. (2003). An evolutionary analysis of orphan genes in *Drosophila*. Genome Res..

[38] Deora A., Gossen B.D., McDonald M.R. (2012). Infection and development of *Plasmodiophora brassicae* in resistant and susceptible canola cultivars. Can. J. Plant Pathol..

[39] Ge Y., Ramchiary N., Wang T., Liang C., Wang N., Wang Z., Choi S.R., Lim Y.P., Piao Z. (2011). Mapping quantitative trait loci for leaf and heading-related traits in Chinese cabbage (*Brassica rapa* L. ssp. *pekinesis*). Hortic. Environ. Biotechnol..

[40] Gu A., Meng C., Chen Y., Wei L., Dong H., Lu Y., Wang Y., Chen X., Zhao J., Shen S. (2017). Coupling Seq-BSA and RNA-Seq analyses reveal the molecular pathway and genes associated with heading type in Chinese Cabbage. Front. Genet..

[41] Liu S., Liu Y., Yang X., Tong C., Edwards D., Parkin I.A.P., Zhao M., Ma J., Yu J., Huang S. (2014). The *Brassica oleracea* genome reveals the asymmetrical evolution of polyploid genomes. Nat. Commun..

[42] Waminal N.E., Perumal S., Lee J., Kim H.H., Yang T.-J. (2016). Repeat Evolution in *Brassica rapa* (AA), *B. oleracea* (CC), and *B. napus* (AACC) Genomes. Plant Breed. Biotechnol..

[43] Carvunis A.R., Rolland T., Wapinski I., Calderwood M.A., Yildirim M.A., Simonis N., Charloteaux B., Hidalgo C.A., Barbette J., Santhanam B. (2012). Proto-genes and de novo gene birth. Nature.

[44] Neme R., Tautz D. (2013). Phylogenetic patterns of emergence of new genes support a model of frequent de novo evolution. BMC Genom..

[45] Zou M., Guo B., He S. (2011). The roles and evolutionary patterns of intronless genes in deuterostomes. Comp. Funct. Genom..

[46] Tautz D., Neme R., Domazet-Lošo T., Maccarrone M. (2013). Evolutionary Origin of Orphan Genes.

[47] Yang J., Liu D., Wang X., Ji C., Cheng F., Liu B., Hu Z., Chen S., Pental D., Ju Y. (2016). The genome sequence of allopolyploid *Brassica juncea* and analysis of differential homoeolog gene expression influencing selection. Nat. Genet..

[48] Chalhoub B., Denoeud F., Liu S., Parkin I.A., Tang H., Wang X., Chiquet J., Belcram H., Tong C., Samans B. (2014). Early allopolyploid evolution in the post-Neolithic *Brassica napus* oilseed genome. Science.

[49] Yang T.J., Kim J.S., Kwon S.J., Lim K.B., Choi B.S., Kim J.A., Jin M., Park J.Y., Lim M.H., Kim H.I. (2006). Sequence-level analysis of the diploidization process in the triplicated *FLOWERING LOCUS C* region of *Brassica rapa*. Plant Cell.

[50] Navabi Z.-K., Huebert T., Sharpe A.G., O’Neill C.M., Bancroft I., Parkin I.A.P. (2013). Conserved microstructure of the *Brassica* B Genome of *Brassica nigra* in relation to homologous regions of *Arabidopsis thaliana*, *B. rapa* and *B. oleracea*. BMC Genom..

[51] Bosch T.C.G., Augustin R., Anton-Erxleben F., Fraune S., Hemmrich G., Zill H., Rosenstiel P., Jacobs G., Schreiber S., Leippe M. (2009). Uncovering the evolutionary history of innate immunity: The simple metazoan *Hydra* uses epithelial cells for host defence. Dev. Comp. Immunol..

[52] Guo W.J., Li P., Ling J., Ye S.P. (2007). Significant comparative characteristics between orphan and nonorphan genes in the rice (*Oryza sativa* L.) genome. Comp. Funct. Genom..

[53] Jiang M., Liu J., Quan X., Quan L., Wu S. (2016). Different chilling stresses stimulated the accumulation of different types of ginsenosides in *Panax ginseng* cells. Acta Physiol. Plant..

[54] Voorrips R.E. (2002). MapChart: Software for the graphical presentation of linkage maps and QTLs. J. Hered..

[55] Pang W., Li X., Choi S., Dhandapani V., Im S., Park M., Soon Jang C., Yang M.-S., Ki Ham I., Mo Lee E. (2015). Development of a leafy *Brassica rapa* fixed line collection for genetic diversity and population structure analysis. Mol. Breed..

[56] Trapnell C., Pachter L., Salzberg S.L. (2009). TopHat: Discovering splice junctions with RNA-Seq. Bioinformatics.

[57] Blankenberg D., Gordon A., Von Kuster G., Coraor N., Taylor J., Nekrutenko A., Galaxy T. (2010). Manipulation of FASTQ data with Galaxy. Bioinformatics.

[58] Trapnell C., Williams B.A., Pertea G., Mortazavi A., Kwan G., van Baren M.J., Salzberg S.L., Wold B.J., Pachter L. (2010). Transcript assembly and quantification by RNA-Seq reveals unannotated transcripts and isoform switching during cell differentiation. Nat. Biotechnol..

[59] Chen J., Piao Y., Liu Y., Li X., Piao Z. (2018). Genome-wide identification and expression analysis of chitinase gene family in *Brassica rapa* reveals its role in clubroot resistance. Plant Sci..

