# Supplementary material for: Mining of Brassica-Specific Genes (BSGs) and Their Induction in Different Developmental Stages and under Plasmodiophora brassicae Stress in Brassica rapa
Source: Int J Mol Sci. 2018 Jul 16;19(7):2064. doi: 10.3390/ijms19072064 (PMC6073354; doi:10.3390/ijms19072064)
Supplement: Supplementary file 1 [file ijms-19-02064-s001.pdf]

Supplementary Materials

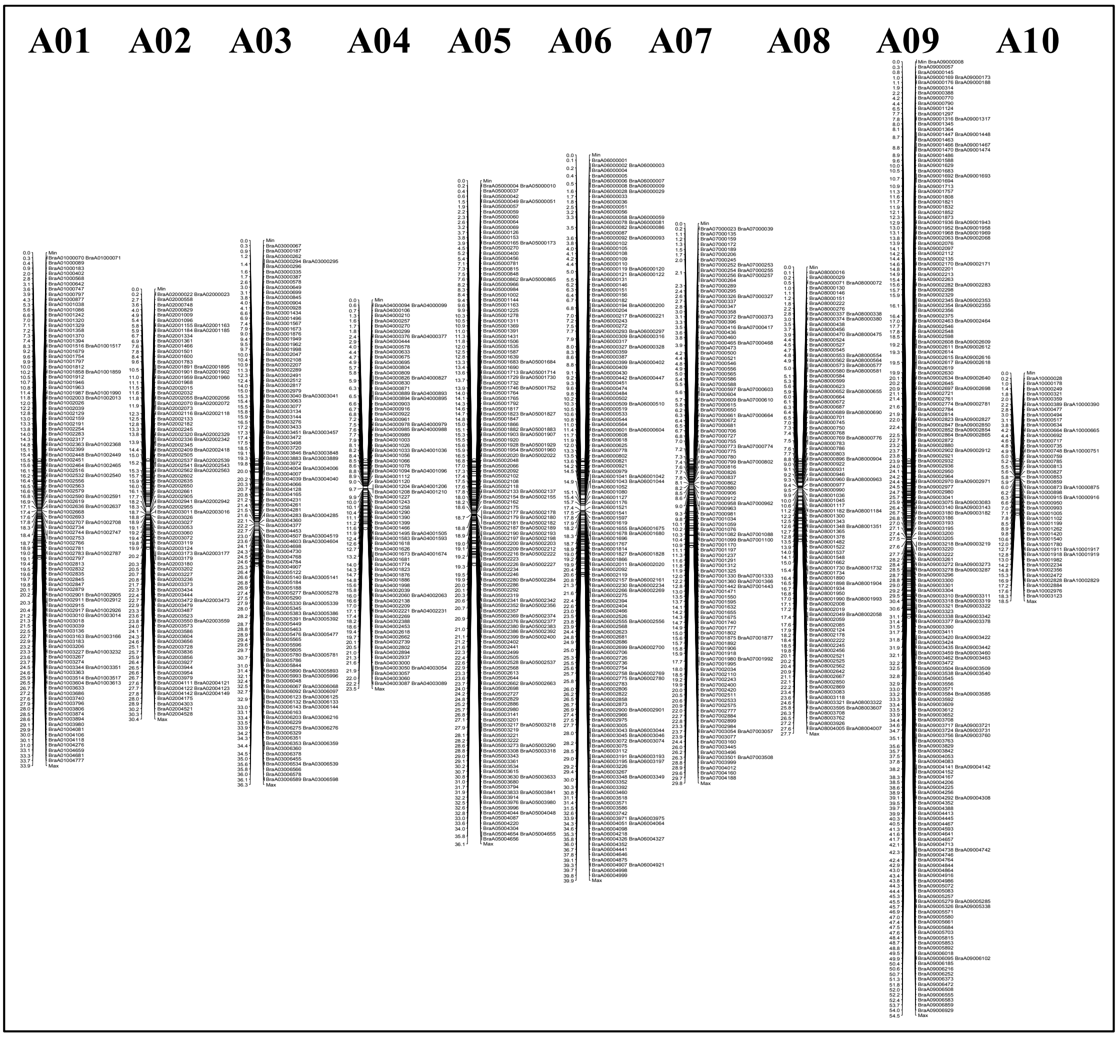

Figure S1. Physical map of *Brassica*-specific genes (BSGs) in each chromosome of the *B. rapa* genome. The BSG physical map was constructed using the chromosomal position of each gene as a starting point along the chromosomes. A01–A10 indicates the 10 chromosomes of the *B. rapa* genome. The white oblong represents chromosome length. Numbers to the left of the chromosome bar indicates the chromosomal position of each BSGs, and words to the right of the chromosome bar represents the identities (IDs) of each BSGs. "Max" refers to the position of the last gene on each chromosome. Genes located on the scaffold were not mapped in this figure.

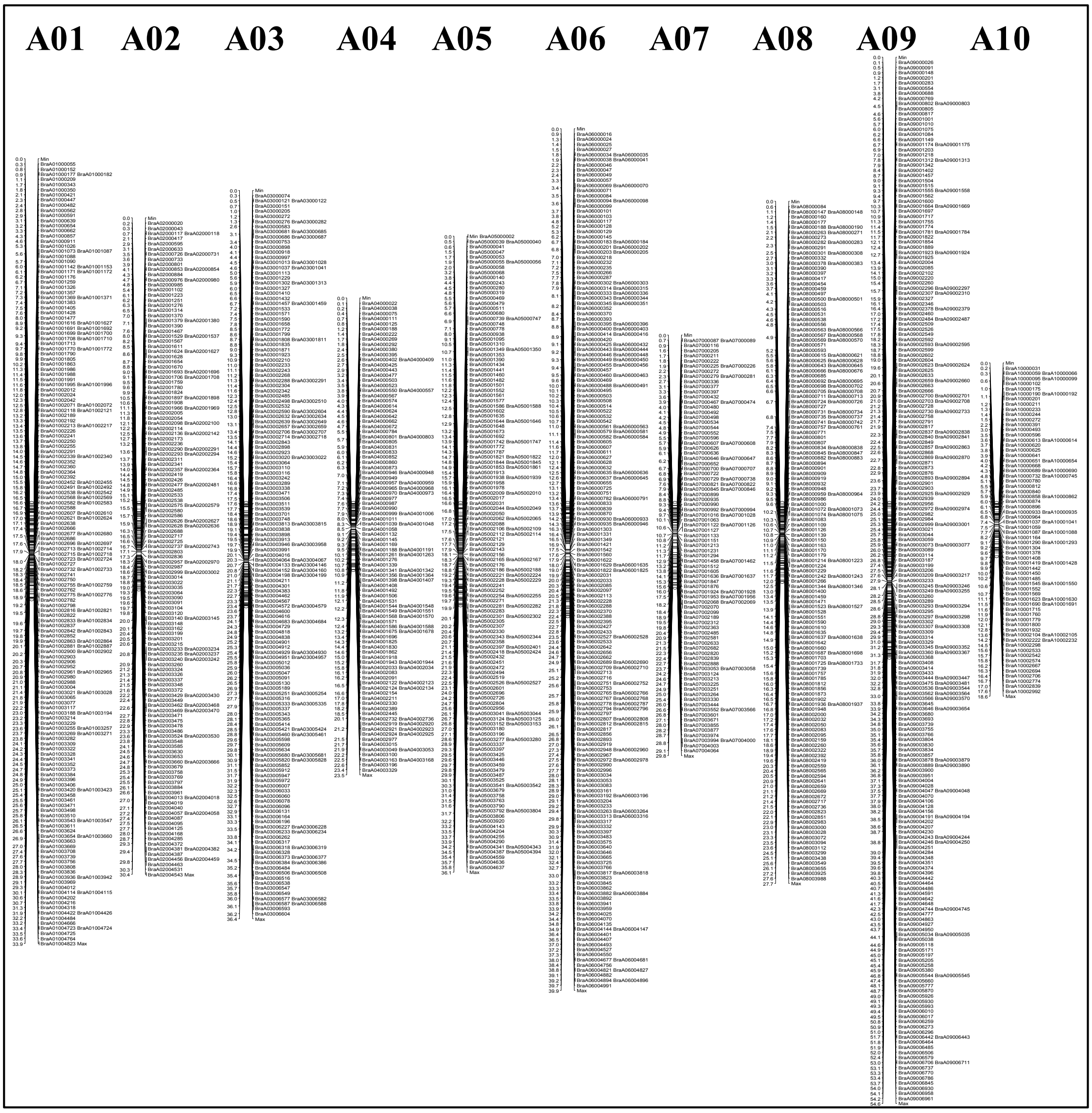

Figure S2. The physical map of Cruciferae-specific genes (CSGs) in each chromosome of *B. rapa* genome.

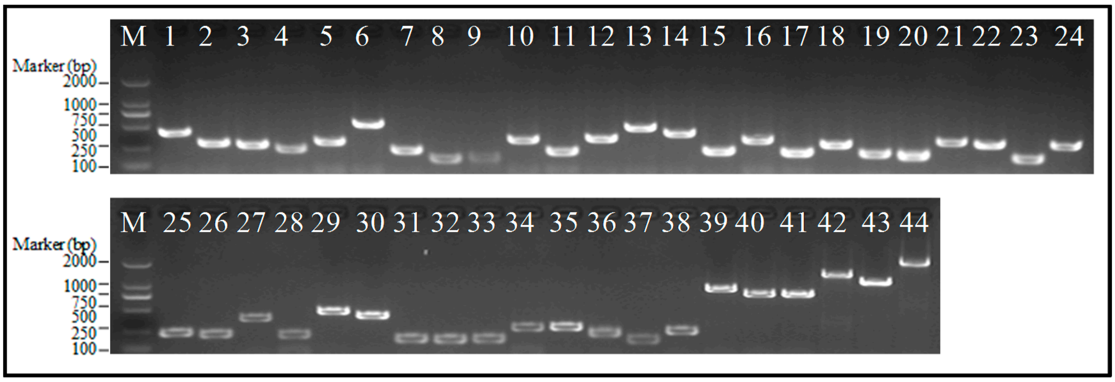

Figure S3. One-hundred and forty-five real A subgenome-specific BSGs verification in Chiifu cultivar *via* PCR assays. Lane numbers 1–44 represent BSGs (see first to third column in Supplementary File 2: Table S3 for more details), and 8 BSGs belong to multiple copy genes, 93 out of 145 BSGs were not detected (third column in Supplementary File 2: Table S3). M indicates the DNA marker.

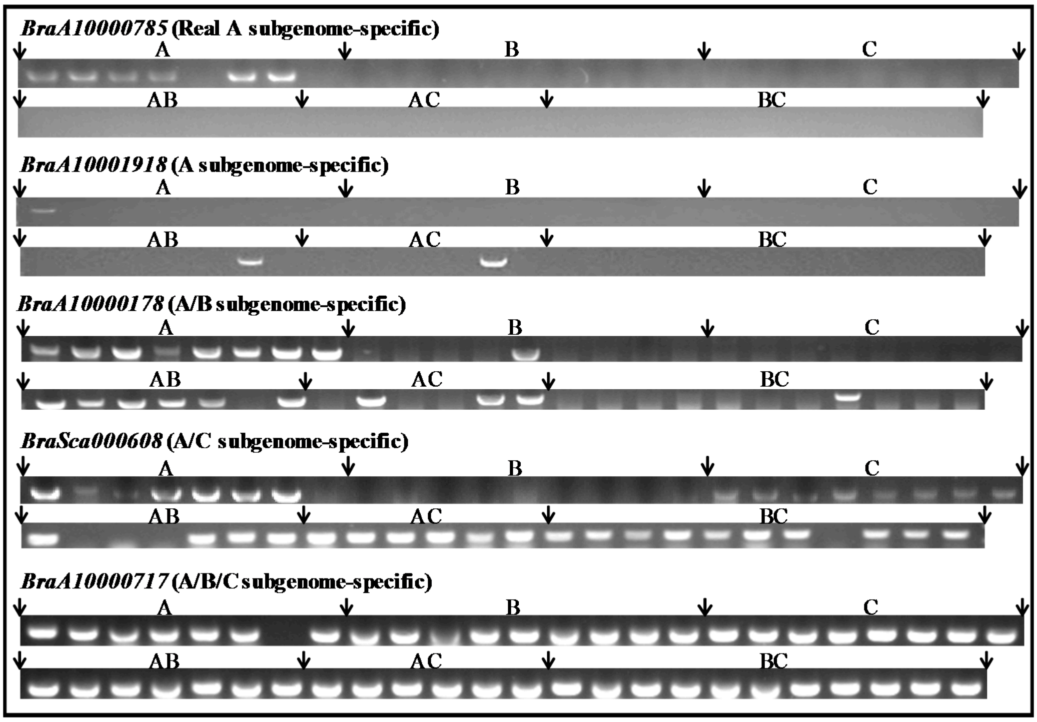

Figure S4. Modules for different types of BSGs in *Brassica* species identified using PCR assays. Details of the remaining amplified BSGs are shown in Supplementary File 2: Table S3. *BraA10000785* belonging to real A subgenome-specific type that only amplified in *B. rapa*. Genes identified in A/B (e.g., *BraA10000178*), A/C (e.g., *BraSca000608*), and A/B/C (e.g., *BraA10000717*) subgenome-specific types also occurred in BC genomes because they contain B and C subgenomes.

Table S1. IDs list of 50 species used for the validation of BSGs.

Species	Numbers	IDs
B. rapa	8	BJN3-2, C24-2, 16ACR73, 16ACR3-1, 16ACR3-2, 16ACR20, 16AJQ15, 16AERMD26B
B. nigra	9	25049, 27060, 27061, 27062, 27063, 27064, 28227, 28228, 28229
B. oleracea	8	QS7, SY-1, LY-3, ECD11, ECD13, ECD15, WJQ, WBS
B. juncea	7	25019, 25078, 26070, 26071, 26072, 27118, 27154
B. napus	6	16ACR19, 16ACR20, 16ACR21, 16ACR22, 16ACR23, 16ACR90
B. carinata	11	28664, 28665, 28666, 28667, 28668, 28669, 28724, 28726, 28727, 28924, G235-1
A. thaliana	1	ecotype Columbia (Col-0)

Table S2. Validation results of BSGs in Chiifu, *A. thaliana*, and other *Brassica* species.

Lane numbers in Fig. S4	Gene IDs*	Reference genome sequencing plant*	Represent plant within Cruciferae*	*Brassica* genomes**						Types of BSGs
		Chiifu	*A. thaliana*	A	B	C	AB	AC	BC	
1	BraA10000785	Y	N	6	0	0	0	0	0	Real A subgenome-specific
2	BraA10001918	Y	N	2	0	0	1	1	0	A subgenome-specific
3	BraSca000020	Y	N	5	0	0	2	4	0	A subgenome-specific
4	BraSca000270	Y	N	5	0	0	4	0	0	A subgenome-specific
5	BraSca000367	Y	N	3	0	0	1	1	0	A subgenome-specific
6	BraSca000627	Y	N	4	0	0	1	4	0	A subgenome-specific
7	BraSca001039	Y	N	7	0	0	2	2	0	A subgenome-specific
8	BraA09001629	Y	N	7	0	0	1	3	0	A subgenome-specific
9	BraA07000615 & BraA09001463	Y	N	7	0	0	3	3	0	A subgenome-specific
10	BraSca000445 & BraA09004053 & BraA05003308	Y	N	6	0	0	1	3	0	A subgenome-specific
11	BraSca001271 & BraA09003195	Y	N	6	0	0	0	2	0	A subgenome-specific
12	BraA10000249	Y	N	6	2	0	7	5	2	A/B subgenome-specific
13	BraA10000359	Y	N	8	1	0	5	4	1	A/B subgenome-specific
14	BraA10000477	Y	N	8	2	0	3	2	0	A/B subgenome-specific
15	BraSca000143	Y	N	8	2	0	7	6	2	A/B subgenome-specific
16	BraSca000146	Y	N	6	3	0	6	1	1	A/B subgenome-specific
17	BraSca000289	Y	N	8	4	0	7	6	1	A/B subgenome-specific
18	BraSca000441	Y	N	8	1	0	7	6	0	A/B subgenome-specific
19	BraSca000661	Y	N	8	5	0	6	3	3	A/B subgenome-specific
20	BraSca001249	Y	N	8	4	0	6	5	5	A/B subgenome-specific

21	BraSca000608	Y	N	7	0	8	4	6	10	A/C subgenome-specific
22	BraSca000334 & BraA03000262	Y	N	7	0	6	4	2	1	A/C subgenome-specific
23	BraA10000717	Y	N	7	9	8	7	6	11	A/B/C subgenome-specific
24	BraA10002884	Y	N	8	4	3	7	6	2	A/B/C subgenome-specific
25	BraSca000160	Y	N	8	8	7	6	5	10	A/B/C subgenome-specific
26	BraSca000205	Y	N	8	9	8	7	6	11	A/B/C subgenome-specific
27	BraSca000216	Y	N	8	9	8	7	6	11	A/B/C subgenome-specific
28	BraSca000335	Y	N	8	9	8	7	6	11	A/B/C subgenome-specific
29	BraSca000346	Y	N	2	2	8	4	6	10	A/B/C subgenome-specific
30	BraSca000423	Y	N	8	2	4	7	6	4	A/B/C subgenome-specific
31	BraSca000476	Y	N	8	9	8	7	6	11	A/B/C subgenome-specific
32	BraSca000502	Y	N	8	9	8	7	6	11	A/B/C subgenome-specific
33	BraSca000553	Y	N	8	6	8	7	6	11	A/B/C subgenome-specific
34	BraSca000870	Y	N	8	9	8	7	6	11	A/B/C subgenome-specific

35	BraSca001087	Y	N	8	3	4	6	6	10	A/B/C subgenome-specific
36	BraSca001291	Y	N	8	4	8	7	6	11	A/B/C subgenome-specific
37	BraA04003089	Y	N	8	9	8	7	6	11	A/B/C subgenome-specific
38	BraSca001287 & BraA09002618 & BraSca000945	Y	N	8	9	8	7	6	11	A/B/C subgenome-specific
39	BraA10000178	Y	N	8	1	0	6	3	1	A/B subgenome-specific
40	BraSca000544	Y	N	7	3	0	7	5	1	A/B subgenome-specific
41	BraSca000578	Y	N	6	3	0	6	6	4	A/B subgenome-specific
42	BraA10001919	Y	N	1	0	0	1	1	0	A subgenome-specific
43	BraA10000759	Y	N	6	1	0	7	5	0	A/B subgenome-specific
44	BraSca001097 & BraA05003841	Y	N	2	0	5	4	6	0	A/C subgenome-specific
/	BraA09001316	N	N	0	0	0	0	0	0	/
/	BraA09001317	N	N	0	0	0	0	0	0	/
/	BraA10000028	N	N	0	0	0	0	0	0	/
/	BraA10000827	N	N	0	0	0	0	0	0	/
/	BraA10001262	N	N	0	0	0	0	0	0	/
/	BraA10001420	N	N	0	0	0	0	0	0	/
/	BraA10002472	N	N	0	0	0	0	0	0	/
/	BraA10002976	N	N	0	0	0	0	0	0	/
/	BraSca000038	N	N	0	0	0	0	0	0	/
/	BraSca000039	N	N	0	0	0	0	0	0	/
/	BraSca000068	N	N	0	0	0	0	0	0	/
/	BraSca000088	N	N	0	0	0	0	0	0	/

/	BraSca000108	N	N	0	0	0	0	0	0	/
/	BraSca000214	N	N	0	0	0	0	0	0	/
/	BraSca000215	N	N	0	0	0	0	0	0	/
/	BraSca000222	N	N	0	0	0	0	0	0	/
/	BraSca000301	N	N	0	0	0	0	0	0	/
/	BraSca000302	N	N	0	0	0	0	0	0	/
/	BraSca000303	N	N	0	0	0	0	0	0	/
/	BraSca000480	N	N	0	0	0	0	0	0	/
/	BraSca000481	N	N	0	0	0	0	0	0	/
/	BraSca000527	N	N	0	0	0	0	0	0	/
/	BraSca000623	N	N	0	0	0	0	0	0	/
/	BraSca000630	N	N	0	0	0	0	0	0	/
/	BraSca000632	N	N	0	0	0	0	0	0	/
/	BraSca000633	N	N	0	0	0	0	0	0	/
/	BraSca000634	N	N	0	0	0	0	0	0	/
/	BraSca000635	N	N	0	0	0	0	0	0	/
/	BraSca000636	N	N	0	0	0	0	0	0	/
/	BraSca000642	N	N	0	0	0	0	0	0	/
/	BraSca000643	N	N	0	0	0	0	0	0	/
/	BraSca000647	N	N	0	0	0	0	0	0	/
/	BraSca000648	N	N	0	0	0	0	0	0	/
/	BraSca000686	N	N	0	0	0	0	0	0	/
/	BraSca000687	N	N	0	0	0	0	0	0	/
/	BraSca000726	N	N	0	0	0	0	0	0	/
/	BraSca000729	N	N	0	0	0	0	0	0	/
/	BraSca000752 & BraSca000754	N	N	0	0	0	0	0	0	/

/	BraSca000757	N	N	0	0	0	0	0	0	/
/	BraSca000758	N	N	0	0	0	0	0	0	/
/	BraSca000768	N	N	0	0	0	0	0	0	/
/	BraSca000811	N	N	0	0	0	0	0	0	/
/	BraSca000840	N	N	0	0	0	0	0	0	/
/	BraSca000842	N	N	0	0	0	0	0	0	/
/	BraSca000849	N	N	0	0	0	0	0	0	/
/	BraSca000878	N	N	0	0	0	0	0	0	/
/	BraSca000879	N	N	0	0	0	0	0	0	/
/	BraSca000880	N	N	0	0	0	0	0	0	/
/	BraSca000881	N	N	0	0	0	0	0	0	/
/	BraSca000882	N	N	0	0	0	0	0	0	/
/	BraSca000883	N	N	0	0	0	0	0	0	/
/	BraSca000884	N	N	0	0	0	0	0	0	/
/	BraSca000902	N	N	0	0	0	0	0	0	/
/	BraSca000910	N	N	0	0	0	0	0	0	/
/	BraSca000914	N	N	0	0	0	0	0	0	/
/	BraSca000915	N	N	0	0	0	0	0	0	/
/	BraSca000929	N	N	0	0	0	0	0	0	/
/	BraSca000930	N	N	0	0	0	0	0	0	/
/	BraSca000932	N	N	0	0	0	0	0	0	/
/	BraSca000933	N	N	0	0	0	0	0	0	/
/	BraSca000936	N	N	0	0	0	0	0	0	/
/	BraSca001006	N	N	0	0	0	0	0	0	/
/	BraSca001077	N	N	0	0	0	0	0	0	/
/	BraSca001082	N	N	0	0	0	0	0	0	/

/	BraSca001101	N	N	0	0	0	0	0	0	/
/	BraSca001121	N	N	0	0	0	0	0	0	/
/	BraSca001125	N	N	0	0	0	0	0	0	/
/	BraSca001133	N	N	0	0	0	0	0	0	/
/	BraSca001134	N	N	0	0	0	0	0	0	/
/	BraSca001135	N	N	0	0	0	0	0	0	/
/	BraSca001145	N	N	0	0	0	0	0	0	/
/	BraSca001146	N	N	0	0	0	0	0	0	/
/	BraSca001150	N	N	0	0	0	0	0	0	/
/	BraSca001191	N	N	0	0	0	0	0	0	/
/	BraSca001193	N	N	0	0	0	0	0	0	/
/	BraSca001199	N	N	0	0	0	0	0	0	/
/	BraSca001208	N	N	0	0	0	0	0	0	/
/	BraSca001257	N	N	0	0	0	0	0	0	/
/	BraSca001267	N	N	0	0	0	0	0	0	/
/	BraSca001275	N	N	0	0	0	0	0	0	/
/	BraSca001278	N	N	0	0	0	0	0	0	/
/	BraSca001288	N	N	0	0	0	0	0	0	/
/	BraSca001294	N	N	0	0	0	0	0	0	/
/	BraSca001297	N	N	0	0	0	0	0	0	/
/	BraSca001299	N	N	0	0	0	0	0	0	/
/	BraSca001301	N	N	0	0	0	0	0	0	/
/	BraSca001302	N	N	0	0	0	0	0	0	/
/	BraSca001303	N	N	0	0	0	0	0	0	/
/	BraSca001304	N	N	0	0	0	0	0	0	/
/	BraSca001305	N	N	0	0	0	0	0	0	/

/	BraSca001307	N	N	0	0	0	0	0	0	/
/	BraSca001309	N	N	0	0	0	0	0	0	/

*"&" in second column indicates the gene belonging to multiple copy genes. "Y" and "N" in third and fourth column indicate that target bands of BSGs can or cannot be amplified in Chiifu and *A. thaliana*, respectively.

**The counts in the 5th to 10th columns represent the numbers of species that can amplify the target band in *B. rapa* (A genome), *B. nigra* (B genome), *B. oleracea* (C genome), *B. juncea* (AB genomes), *B. napus* (AC genomes), and *B. carinata* (BC genomes).

Table S3. Results of BSGs protein sequence searched against EST sequences *via* TBLASTN.

Gene IDs	Accessions	Identity (%)	Query cover (%)	q_start	q_end	s_start	s_end	evalue	Descriptions
BraA07000615	EX118638.1	77	100	1	52	207	52	6.54E-19	BR102468 cotyledon cDNA library KHCT *Brassica rapa* subsp. *pekinensis* cDNA clone KHCT-16C08 5', mRNA sequence
BraA07000615	EX068531.1	77	100	1	52	232	77	9.50E-19	BR053175 seedling cDNA library KBPS *Brassica rapa* subsp. *pekinensis* cDNA clone KBPS-007C08 5', mRNA sequence
BraA07000615	EX126481.1	77	100	1	52	328	173	1.20E-18	BR110311 etiolated mature leaf cDNA library KHLW *Brassica rapa* subsp. *pekinensis* cDNA clone KHLW-13G08 5', mRNA sequence
BraA07000615	EX117988.1	77	100	1	52	322	167	1.20E-18	BR101818 cotyledon cDNA library KHCT *Brassica rapa* subsp. *pekinensis* cDNA clone KHCT-09A06 5', mRNA sequence
BraA07000615	EX070668.1	77	100	1	52	270	115	1.50E-18	BR055313 seedling cDNA library KBPS *Brassica rapa* subsp. *pekinensis* cDNA clone KBPS-038E06 5', mRNA sequence
BraA09001463	EX118638.1	77	100	1	52	207	52	6.54E-19	BR102468 cotyledon cDNA library KHCT *Brassica rapa* subsp. *pekinensis* cDNA clone KHCT-16C08 5', mRNA sequence
BraA09001463	EX068531.1	77	100	1	52	232	77	9.50E-19	BR053175 seedling cDNA library KBPS *Brassica rapa* subsp. *pekinensis* cDNA clone KBPS-007C08 5', mRNA sequence
BraA09001463	EX126481.1	77	100	1	52	328	173	1.20E-18	BR110311 etiolated mature leaf cDNA library KHLW *Brassica rapa* subsp. *pekinensis* cDNA clone KHLW-13G08 5', mRNA sequence
BraA09001463	EX117988.1	77	100	1	52	322	167	1.20E-18	BR101818 cotyledon cDNA library KHCT *Brassica rapa* subsp. *pekinensis* cDNA clone KHCT-09A06 5', mRNA sequence
BraA09001463	EX070668.1	77	100	1	52	270	115	1.50E-18	BR055313 seedling cDNA library KBPS *Brassica rapa* subsp. *pekinensis* cDNA clone KBPS-038E06 5', mRNA sequence
BraA09001629	EX041676.1	76	100	1	59	22	198	7.05E-25	BR026320 floral bud cDNA library KBFL *Brassica rapa* subsp. *pekinensis* cDNA clone KBFL-094B07 5', mRNA sequence
BraA04003089	EX085504.1	100	75	16	61	688	551	1.19E-24	BR070148 primary inflorescence cDNA library KBST *Brassica rapa* subsp. *pekinensis* cDNA clone KBST-22H03 5', mRNA sequence
BraA03005140	GR719604.1	100	100	1	110	550	221	1.75E-58	MLN1_2440 *Brassica rapa* mature lateral nectary, non-normalized (MLN1) *Brassica rapa* cDNA, mRNA sequence
BraA03005141	GR719604.1	100	100	1	83	469	221	4.18E-37	MLN1_2440 *Brassica rapa* mature lateral nectary, non-normalized (MLN1) *Brassica rapa* cDNA, mRNA sequence
BraA09003504	CX265965.1	98	100	16	62	172	312	9.15E-25	38RDBRM_UP_005_D07_30MAR2004_057 *Brassica rapa* 38RDBRM *Brassica rapa* cDNA 5', mRNA sequence
BraA03005596	EX048271.1	82	75	1	51	127	279	4.64E-22	BR032915 floral buds cDNA library KBFS *Brassica rapa* subsp. *pekinensis* cDNA clone KBFS-030B11 5', mRNA sequence
BraA03005605	EX110857.1	100	100	1	64	76	267	2.67E-38	BR097147 whole plant cDNA library KFYP *Brassica rapa* subsp. *pekinensis* cDNA clone KFYP-067D07 5', mRNA sequence
BraA07000755	EX051846.1	93	99	1	120	366	4	9.65E-75	BR036490 floral buds cDNA library KBFS *Brassica rapa* subsp. *pekinensis* cDNA clone KBFS-078H06 5', mRNA sequence
BraA07000755	EX110271.1	94	80	24	121	869	573	3.57E-57	BR096561 whole plant cDNA library KFYP *Brassica rapa* subsp. *pekinensis* cDNA clone KFYP-058G10 5', mRNA sequence
BraA09003322	EX139554.1	78	100	1	63	211	399	9.81E-24	BR123384 primary leaf cDNA library KLPS *Brassica rapa* subsp. *pekinensis* cDNA clone KLPS-14G08 5', mRNA sequence
BraA03003276	EX051846.1	100	99	1	121	366	4	3.15E-84	BR036490 floral buds cDNA library KBFS *Brassica rapa* subsp. *pekinensis* cDNA clone KBFS-078H06 5', mRNA sequence

BraA03003276	EX110271.1	100	81	24	122	869	573	6.15E-65	BR096561 whole plant cDNA library KFYP *Brassica rapa* subsp. *pekinensis* cDNA clone KFYP-058G10 5', mRNA sequence
BraA03004039	EX061422.1	100	100	1	91	468	196	4.01E-61	BR046066 salt-treated whole plant cDNA library KBLS *Brassica rapa* subsp. *pekinensis* cDNA clone KBLS-095G04 5', mRNA sequence
BraA03004039	EX113420.1	100	85	14	91	544	311	6.29E-51	BR099710 whole plant cDNA library KFYP *Brassica rapa* subsp. *pekinensis* cDNA clone KFYP-103C02 5', mRNA sequence
BraA03004040	EX061422.1	75	83	14	81	688	485	2.30E-24	BR046066 salt-treated whole plant cDNA library KBLS *Brassica rapa* subsp. *pekinensis* cDNA clone KBLS-095G04 5', mRNA sequence
BraA06003586	EX045997.1	100	74	18	109	97	372	8.07E-64	BR030641 floral bud cDNA library KBFL *Brassica rapa* subsp. *pekinensis* cDNA clone KBFL-152E05 5', mRNA sequence
BraA06003586	CX268052.1	100	74	18	109	151	426	9.42E-64	38RDBRM_UP_030_B02_12JUL2004_014 *Brassica rapa* 38RDBRM *Brassica rapa* cDNA 5', mRNA sequence
BraA06003586	EX045580.1	100	74	18	109	89	364	1.00E-63	BR030224 floral bud cDNA library KBFL *Brassica rapa* subsp. *pekinensis* cDNA clone KBFL-147B07 5', mRNA sequence
BraA06003571	EX087170.1	99	71	1	250	79	828	0	BR073460 flower cDNA library KFFB *Brassica rapa* subsp. *pekinensis* cDNA clone KFFB-025B01 5', mRNA sequence
BraA06003518	EX027694.1	78	90	22	71	412	263	1.61E-19	BR012338 callus cDNA library KBCG *Brassica rapa* subsp. *pekinensis* cDNA clone KBCG-050E12 5', mRNA sequence
BraA06003518	EX035547.1	78	90	22	71	412	263	1.70E-19	BR020191 callus cDNA library KBCG *Brassica rapa* subsp. *pekinensis* cDNA clone KBCG-173D02 5', mRNA sequence
BraA06003518	EX027961.1	78	90	22	71	412	263	2.08E-19	BR012605 callus cDNA library KBCG *Brassica rapa* subsp. *pekinensis* cDNA clone KBCG-054B10 5', mRNA sequence
BraA01003686	EX086139.1	82	75	26	103	3	236	2.04E-37	BR072429 flower cDNA library KFFB *Brassica rapa* subsp. *pekinensis* cDNA clone KFFB-008A12 5', mRNA sequence
BraA07001366	EX081849.1	89	74	6	69	61	252	3.81E-34	BR066493 silique cDNA library KBSP *Brassica rapa* subsp. *pekinensis* cDNA clone KBSP-068C09 5', mRNA sequence
BraA07001366	EX081997.1	89	74	6	69	50	241	7.8E-34	BR066641 silique cDNA library KBSP *Brassica rapa* subsp. *pekinensis* cDNA clone KBSP-070C03 5', mRNA sequence
BraA07001366	GR724092.1	89	74	6	69	153	344	5.30E-33	MMN1_1533 *Brassica rapa* mature median nectary, non-normalized (MMN1) *Brassica rapa* cDNA similar to AGP9 (ARABINOGALACTAN PROTEIN 9), mRNA sequence
BraA07001366	FY069560.1	89	74	6	69	365	174	6.97E-33	FY069560 Chinese cabbage Full-length cDNA library *Brassica rapa* subsp. *pekinensis* cDNA clone BBRAF04F22 3', mRNA sequence
BraA07001366	FY064911.1	89	74	6	69	160	351	9.36E-33	FY064911 Chinese cabbage Full-length cDNA library *Brassica rapa* subsp. *pekinensis* cDNA clone BBRAF04F22 5', mRNA sequence
BraA07001366	EX082109.1	89	70	6	66	50	232	2.27E-31	BR066753 silique cDNA library KBSP *Brassica rapa* subsp. *pekinensis* cDNA clone KBSP-071E12 5', mRNA sequence
BraA07001366	FY426962.1	77	70	6	66	340	158	4.39E-26	FY426962 Chinese cabbage Full-length cDNA library *Brassica rapa* subsp. *pekinensis* cDNA clone BBRAF41N21 3', mRNA sequence
BraA07001366	FY422021.1	77	70	6	66	137	319	5.96E-26	FY422021 Chinese cabbage Full-length cDNA library *Brassica rapa* subsp. *pekinensis* cDNA clone BBRAF41N21 5', mRNA sequence
BraA07001366	FY426837.1	77	70	6	66	376	194	7.83E-26	FY426837 Chinese cabbage Full-length cDNA library *Brassica rapa* subsp. *pekinensis* cDNA clone BBRAF41I12 3', mRNA sequence
BraA07001366	FY421896.1	77	70	6	66	183	365	1.04E-25	FY421896 Chinese cabbage Full-length cDNA library *Brassica rapa* subsp. *pekinensis* cDNA clone BBRAF41I12 5', mRNA sequence
BraA03005780	EX037210.1	100	100	1	131	71	463	4.38E-90	BR021854 floral bud cDNA library KBFL *Brassica rapa* subsp. *pekinensis* cDNA clone KBFL-020H09 5', mRNA sequence
BraA03005780	EX091532.1	100	100	1	131	35	427	4.53E-90	BR077822 flower cDNA library KFFB *Brassica rapa* subsp. *pekinensis* cDNA clone KFFB-089H11 5', mRNA sequence
BraA01003018	EX109939.1	85	100	1	109	225	551	1.62E-78	BR096229 whole plant cDNA library KFYP *Brassica rapa* subsp. *pekinensis* cDNA clone KFYP-054B06 5', mRNA sequence

BraA01003018	EX109939.1	85	76	108	177	619	828	1.62E-78	BR074101 flower cDNA library KFFB *Brassica rapa* subsp. *pekinensis* cDNA clone KFFB-034G03 5', mRNA sequence
BraA08000151	EX134469.1	100	100	1	132	679	284	1.53E-92	BR118299 root cDNA library KHRT *Brassica rapa* subsp. *pekinensis* cDNA clone KHRT-46E11 5', mRNA sequence
BraA08000151	EX133432.1	100	100	1	132	683	288	1.66E-92	BR117262 root cDNA library KHRT *Brassica rapa* subsp. *pekinensis* cDNA clone KHRT-33F09 5', mRNA sequence
BraA08000151	EX092261.1	100	100	1	132	725	330	1.83E-92	BR078551 flower cDNA library KFFB *Brassica rapa* subsp. *pekinensis* cDNA clone KFFB-102C11 5', mRNA sequence
BraA08000151	EX093316.1	100	100	1	132	707	312	3.60E-92	BR079606 flower cDNA library KFFB *Brassica rapa* subsp. *pekinensis* cDNA clone KFFB-120E01 5', mRNA sequence
BraA08000151	EX127735.1	98	86	19	132	526	185	4.29E-75	BR111565 etiolated mature leaf cDNA library KHLW *Brassica rapa* subsp. *pekinensis* cDNA clone KHLW-29B08 5', mRNA sequence
BraA08000151	EX136763.1	100	100	1	77	670	440	9.66E-68	BR120593 root cDNA library KHRT *Brassica rapa* subsp. *pekinensis* cDNA clone KHRT-75F07 5', mRNA sequence
BraA08000151	EX136763.1	100	75	78	132	438	274	9.66E-68	E0533 Chinese cabbage etiolated seedling library *Brassica rapa* subsp. *pekinensis* cDNA clone E0533, mRNA sequence
BraA10003123	CX268325.1	93	87	42	132	314	586	8.18E-57	38RDBRM_UP_033_E05_13JUL2004_039 *Brassica rapa* 38RDBRM *Brassica rapa* cDNA 5', mRNA sequence
BraA07001980	EX088703.1	76	83	1	59	40	216	2.68E-31	BR074993 flower cDNA library KFFB *Brassica rapa* subsp. *pekinensis* cDNA clone KFFB-052A05 5', mRNA sequence
BraA01002448	EX101288.1	100	78	21	94	503	724	3.59E-46	BR087578 root cDNA library KFRT *Brassica rapa* subsp. *pekinensis* cDNA clone KFRT-043A02 5', mRNA sequence
BraA01002532	CO749527.1	100	100	1	58	258	85	3.45E-36	KBrAE002E19c A leaf cDNA library of Chinese Cabbage, *Brassica rapa Brassica rapa* subsp. *pekinensis* cDNA clone KBrAE002E19, mRNA sequence
BraA01002532	EX094569.1	100	100	1	58	214	387	2.54E-35	BR080859 infected leaf cDNA library KFPC *Brassica rapa* subsp. *pekinensis* cDNA clone KFPC-002D08 5', mRNA sequence
BraA01002532	EX027764.1	98	100	1	58	19	192	9.23E-34	BR012408 callus cDNA library KBCG *Brassica rapa* subsp. *pekinensis* cDNA clone KBCG-051E11 5', mRNA sequence
BraA01002532	EX035146.1	97	100	1	58	16	189	9.14E-33	BR019790 callus cDNA library KBCG *Brassica rapa* subsp. *pekinensis* cDNA clone KBCG-167D03 5', mRNA sequence
BraA01002532	EX110643.1	98	91	6	58	244	402	1.27E-31	BR096933 whole plant cDNA library KFYP *Brassica rapa* subsp. *pekinensis* cDNA clone KFYP-064D08 5', mRNA sequence
BraA01002532	CO749613.1	88	74	2	44	131	3	2.53E-22	KBrAE002J22c A leaf cDNA library of Chinese Cabbage, *Brassica rapa Brassica rapa* subsp. *pekinensis* cDNA clone KBrAE002J22, mRNA sequence
BraA01002532	EX098391.1	100	70	18	58	3	125	7.66E-22	BR084681 root cDNA library KFRT *Brassica rapa* subsp. *pekinensis* cDNA clone KFRT-003H09 5', mRNA sequence
BraA01002532	EX101476.1	71	87	23	58	881	735	1.49E-13	BR087766 root cDNA library KFRT *Brassica rapa* subsp. *pekinensis* cDNA clone KFRT-045F11 5', mRNA sequence
BraA09006583	EX095964.1	72	100	1	79	261	25	1.86E-28	BR082254 infected leaf cDNA library KFPC *Brassica rapa* subsp. *pekinensis* cDNA clone KFPC-024B12 5', mRNA sequence
BraA09006583	EX097428.1	72	100	1	79	261	25	2.58E-28	BR083718 infected leaf cDNA library KFPC *Brassica rapa* subsp. *pekinensis* cDNA clone KFPC-047H07 5', mRNA sequence
BraA04001674	EX138134.1	86	100	1	56	469	302	1.66E-25	BR121964 root cDNA library KHRT *Brassica rapa* subsp. *pekinensis* cDNA clone KHRT-93D10 5', mRNA sequence
BraA04002060	EX049722.1	72	78	1	72	211	2	2.03E-25	BR034366 floral buds cDNA library KBFS *Brassica rapa* subsp. *pekinensis* cDNA clone KBFS-050C07 5', mRNA sequence
BraA04002063	EX049722.1	72	78	1	72	211	2	2.03E-25	BR034366 floral buds cDNA library KBFS *Brassica rapa* subsp. *pekinensis* cDNA clone KBFS-050C07 5', mRNA sequence
BraA07000468	EX046052.1	100	95	1	70	440	649	6.18E-44	BR030696 floral bud cDNA library KBFL *Brassica rapa* subsp. *pekinensis* cDNA clone KBFL-153B12 5', mRNA sequence
BraA05002292	EX100350.1	70	96	1	105	364	810	1.40E-64	BR086640 root cDNA library KFRT *Brassica rapa* subsp. *pekinensis* cDNA clone KFRT-030D09 5', mRNA sequence

BraA05002383	EX017351.1	76	90	40	77	570	683	2.05E-21	BR001995 light-chilled whole plant cDNA library KBCD *Brassica rapa* subsp. *pekinensis* cDNA clone KBCD-003B01 5', mRNA sequence
BraA05002383	ES932277.1	92	100	41	77	192	82	7.50E-17	37RDBRH_UP_083_A08_23JAN2006_064 *Brassica rapa* 37RDBRH *Brassica rapa* cDNA 5', mRNA sequence
BraA05003534	GR726430.1	78	78	1	63	314	502	1.06E-27	MMN2_1046 *Brassica rapa* mature median nectary, normalized (MMN2) *Brassica rapa* cDNA, mRNA sequence
BraA07002575	GR722474.1	76	74	1	55	204	368	8.39E-21	MLN2_2496 *Brassica rapa* mature lateral nectary, normalized (MLN2) *Brassica rapa* cDNA similar to leucine-rich repeat transmembrane protein kinase, putative, mRNA sequence
BraA02004142	EX094491.1	93	100	1	143	679	251	5.38E-91	BR080781 infected leaf cDNA library KFPC *Brassica rapa* subsp. *pekinensis* cDNA clone KFPC-001D08 5', mRNA sequence
BraA02004142	EX098295.1	90	76	35	143	527	201	2.02E-64	BR084585 root cDNA library KFRT *Brassica rapa* subsp. *pekinensis* cDNA clone KFRT-002F04 5', mRNA sequence
BraA02003944	GR719604.1	81	100	1	110	550	221	3.59E-55	MLN1_2440 *Brassica rapa* mature lateral nectary, non-normalized (MLN1) *Brassica rapa* cDNA, mRNA sequence
BraA09004225	GR726430.1	78	78	1	63	314	502	1.06E-27	MMN2_1046 *Brassica rapa* mature median nectary, normalized (MMN2) *Brassica rapa* cDNA, mRNA sequence
BraA09004141	EE529217.1	100	100	1	62	33	218	2.20E-38	39RDBRT_UP_056_G01_30NOV2005_003 *Brassica rapa* 39RDBRT *Brassica rapa* cDNA 5', mRNA sequence
BraA08003083	EX041755.1	100	100	1	111	175	507	1.08E-73	BR026399 floral bud cDNA library KBFL *Brassica rapa* subsp. *pekinensis* cDNA clone KBFL-095B08 5', mRNA sequence
BraA08003083	EX100555.1	100	100	1	111	539	207	1.31E-73	BR086845 root cDNA library KFRT *Brassica rapa* subsp. *pekinensis* cDNA clone KFRT-033A07 5', mRNA sequence
BraA08003083	EX107779.1	100	100	1	111	587	255	1.68E-73	BR094069 whole plant cDNA library KFYP *Brassica rapa* subsp. *pekinensis* cDNA clone KFYP-019H07 5', mRNA sequence
BraA08003083	FY069958.1	99	100	1	111	167	499	2.11E-73	FY069958 Chinese cabbage Full-length cDNA library *Brassica rapa* subsp. *pekinensis* cDNA clone BBRAF05I12 3', mRNA sequence
BraA08003083	FY065327.1	99	100	1	111	564	232	2.11E-73	FY065327 Chinese cabbage Full-length cDNA library *Brassica rapa* subsp. *pekinensis* cDNA clone BBRAF05I12 5', mRNA sequence
BraA08003083	FY420764.1	99	100	1	111	641	309	3.95E-73	FY420764 Chinese cabbage Full-length cDNA library *Brassica rapa* subsp. *pekinensis* cDNA clone BBRAF38G10 5', mRNA sequence
BraA08003083	FY425725.1	99	100	1	111	198	530	6.48E-73	FY425725 Chinese cabbage Full-length cDNA library *Brassica rapa* subsp. *pekinensis* cDNA clone BBRAF38G10 3', mRNA sequence
BraA08003083	ES934351.1	100	84	18	111	540	259	2.79E-61	38RDBRM_UP_078_H01_18JAN2006_001 *Brassica rapa* 38RDBRM *Brassica rapa* cDNA 5', mRNA sequence
BraA01001358	ES936729.1	100	100	1	72	592	377	2.12E-45	39RDBRT_UP_086_H04_24JAN2006_018 *Brassica rapa* 39RDBRT *Brassica rapa* cDNA 5', mRNA sequence
BraA01001358	ES936673.1	99	100	1	72	542	327	2.42E-45	39RDBRT_UP_086_B09_24JAN2006_077 *Brassica rapa* 39RDBRT *Brassica rapa* cDNA 5', mRNA sequence
BraA01001358	EE528988.1	99	100	1	72	542	327	2.26E-44	39RDBRT_UP_053_A12_30NOV2005_096 *Brassica rapa* 39RDBRT *Brassica rapa* cDNA 5', mRNA sequence
BraA01001358	EE523758.1	100	90	20	72	522	364	2.03E-31	37RDBRH_UP_019_H11_31MAR2004_081 *Brassica rapa* 37RDBRH *Brassica rapa* cDNA 5', mRNA sequence
BraA02001600	DY009744.1	70	100	9	99	133	360	1.78E-33	8RDBRH_UP_012_F06_12SEP2003_038 *Brassica rapa* - 8RDBRH *Brassica rapa* cDNA 5', mRNA sequence
BraA06001678	EX048733.1	100	71	1	46	149	12	3.27E-25	BR033377 floral buds cDNA library KBFS *Brassica rapa* subsp. *pekinensis* cDNA clone KBFS-037B12 5', mRNA sequence
BraA06001678	EX099405.1	100	71	1	46	161	24	3.97E-24	BR085695 root cDNA library KFRT *Brassica rapa* subsp. *pekinensis* cDNA clone KFRT-017E11 5', mRNA sequence
BraA06001680	EX048733.1	100	71	1	46	149	12	3.27E-25	BR033377 floral buds cDNA library KBFS *Brassica rapa* subsp. *pekinensis* cDNA clone KBFS-037B12 5', mRNA sequence

BraA06001680	EX099405.1	100	71	1	46	161	24	3.97E-24	BR085695 root cDNA library KFRT *Brassica rapa* subsp. *pekinensis* cDNA clone KFRT-017E11 5', mRNA sequence
BraA01002013	EX041343.1	82	100	1	97	454	98	1.25E-61	BR025987 floral bud cDNA library KBFL *Brassica rapa* subsp. *pekinensis* cDNA clone KBFL-089H10 5', mRNA sequence
BraA06004327	GR724915.1	78	98	2	70	309	515	6.09E-29	MMN1_2356 *Brassica rapa* mature median nectary, non-normalized (MMN1) *Brassica rapa* cDNA similar to transcription factor, mRNA sequence
BraA10000875	EX092206.1	100	100	1	67	168	368	8.01E-40	BR078496 flower cDNA library KFFB *Brassica rapa* subsp. *pekinensis* cDNA clone KFFB-101D05 5', mRNA sequence
BraA08001993	ES933928.1	95	83	1	65	369	563	1.73E-35	37RDBRH_UP_104_F03_24JAN2006_021 *Brassica rapa* 37RDBRH *Brassica rapa* cDNA 5', mRNA sequence
BraA06002266	DC845503.1	100	100	1	58	200	373	8.10E-37	DC845503 Chinese cabbage cDNA library, 2 weeks old aerial part *Brassica rapa* subsp. *pekinensis* cDNA clone BRest06-M13 5', mRNA sequence
BraA06002266	EX086534.1	100	100	1	58	242	415	2.31E-35	BR072824 flower cDNA library KFFB *Brassica rapa* subsp. *pekinensis* cDNA clone KFFB-013H10 5', mRNA sequence
BraA06002266	EX060056.1	100	81	12	58	133	273	6.18E-28	BR044700 salt-treated whole plant cDNA library KBLS *Brassica rapa* subsp. *pekinensis* cDNA clone KBLS-067A07 5', mRNA sequence
BraA09000145	CX272125.1	100	100	1	58	130	303	7.89E-36	39RDBRT_UP_036_D04_20JUL2004_026 *Brassica rapa* 39RDBRT *Brassica rapa* cDNA 5', mRNA sequence
BraA09000145	CX266372.1	100	100	1	58	136	309	3.11E-35	38RDBRM_UP_009_H09_01APR2004_065 *Brassica rapa* 38RDBRM *Brassica rapa* cDNA 5', mRNA sequence
BraA06004921	CX266372.1	97.5	98	1	40	136	255	2.32E-26	38RDBRM_UP_009_H09_01APR2004_065 *Brassica rapa* 38RDBRM *Brassica rapa* cDNA 5', mRNA sequence
BraA06004921	CX272125.1	98	98	1	40	130	249	2.42E-26	39RDBRT_UP_036_D04_20JUL2004_026 *Brassica rapa* 39RDBRT *Brassica rapa* cDNA 5', mRNA sequence
BraSca000413	EX086238.1	100	72	1	72	423	208	2.75E-44	BR072528 flower cDNA library KFFB *Brassica rapa* subsp. *pekinensis* cDNA clone KFFB-009D02 5', mRNA sequence
BraA09000388	FY418925.1	78	87	19	130	248	583	5.87E-52	FY418925 Chinese cabbage Full-length cDNA library *Brassica rapa* subsp. *pekinensis* cDNA clone BBRAF33F04 5', mRNA sequence
BraA09000388	FY423964.1	78	87	19	130	741	406	7.65E-52	FY423964 Chinese cabbage Full-length cDNA library *Brassica rapa* subsp. *pekinensis* cDNA clone BBRAF33F04 3', mRNA sequence
BraA01001946	EX126596.1	95	82	1	83	4	264	9.19E-53	BR110426 etiolated mature leaf cDNA library KHLW *Brassica rapa* subsp. *pekinensis* cDNA clone KHLW-15C03 5', mRNA sequence
BraA02001891	EX132312.1	100	74	10	80	182	394	3.68E-44	BR116142 root cDNA library KHRT *Brassica rapa* subsp. *pekinensis* cDNA clone KHRT-19D01 5', mRNA sequence
BraA02001891	EX086800.1	100	74	10	80	398	610	5.49E-44	BR073090 flower cDNA library KFFB *Brassica rapa* subsp. *pekinensis* cDNA clone KFFB-017H09 5', mRNA sequence
BraSca001227	EX126426.1	100	100	1	47	13	153	3.65E-25	BR110256 etiolated mature leaf cDNA library KHLW *Brassica rapa* subsp. *pekinensis* cDNA clone KHLW-13A11 5', mRNA sequence

Table S4. Results of BSGs protein sequence searched against PlantGDB-assembled unique transcripts (PUTs) via TBLASTN.

Gene IDs	PUTs IDs	Identity (%)	Query cover (%)	q_start	q_end	s_start	s_end	evalue
BraA01001358	PUT-171a-Brassica_rapa-6004	100	100	1	72	592	377	3.06E-46
BraA01001516	PUT-171a-Brassica_rapa-27803	72	94	1	25	107	175	6.56E-10
BraA01001517	PUT-171a-Brassica_rapa-27803	72	87	1	25	107	175	4.26E-07
BraA01001812	PUT-171a-Brassica_rapa-45173	100	98	27	50	617	688	6.92E-10
BraA01001812	PUT-171a-Brassica_rapa-45173	92	98	1	25	239	313	8.26E-09
BraA01001946	PUT-171a-Brassica_rapa-18100	95	82	1	83	4	264	6.26E-53
BraA01002013	PUT-171a-Brassica_rapa-1941	82	100	1	97	454	98	3.12E-62
BraA01002448	PUT-171a-Brassica_rapa-36458	100	79	21	94	503	724	8.93E-47
BraA01002532	PUT-171a-Brassica_rapa-7406	98	91	6	58	219	377	1.48E-30
BraA01002532	PUT-171a-Brassica_rapa-7453	100	71	18	58	3	125	1.91E-22
BraA01002797	PUT-171a-Brassica_rapa-45073	73	87	14	103	457	191	9.59E-21
BraA01002797	PUT-171a-Brassica_rapa-31692	70	87	14	103	443	177	3.33E-19
BraA01003018	PUT-171a-Brassica_rapa-20942	99	100	1	109	257	583	2.51E-79
BraA01003018	PUT-171a-Brassica_rapa-20942	100	100	108	177	651	860	2.51E-79
BraA01003686	PUT-171a-Brassica_rapa-24106	99	75	26	103	3	236	4.39E-38
BraA02001600	PUT-171a-Brassica_rapa-12853	70	100	9	99	133	360	3.23E-34
BraA02001600	PUT-171a-Brassica_rapa-12853	95	100	1	42	68	193	1.21E-21
BraA02001891	PUT-171a-Brassica_rapa-41797	100	75	10	80	398	610	1.37E-44
BraA02001891	PUT-171a-Brassica_rapa-44466	100	75	10	80	182	394	2.37E-44
BraA02003173	PUT-171a-Brassica_rapa-34790	100	84	14	80	365	565	7.25E-43
BraA02004142	PUT-171a-Brassica_rapa-33473	100	100	1	143	679	251	1.34E-91
BraA03001434	PUT-171a-Brassica_rapa-14386	100	100	1	53	63	221	1.43E-05

BraA03003276	PUT-171a-Brassica_rapa-2970	100	100	1	122	956	591	3.00E-82
BraA03004039	PUT-171a-Brassica_rapa-8252	100	100	1	91	583	311	3.58E-61
BraA03004040	PUT-171a-Brassica_rapa-8252	100	84	14	81	803	600	1.16E-24
BraA03004261	PUT-171a-Brassica_rapa-42545	89	81	3	38	143	250	1.53E-09
BraA03005391	PUT-171a-Brassica_rapa-43719	72	98	38	62	512	438	2.02E-09
BraA03005596	PUT-171a-Brassica_rapa-23289	82	75	1	51	127	279	1.15E-22
BraA03005605	PUT-171a-Brassica_rapa-196	100	100	1	64	76	267	6.66E-39
BraA03005780	PUT-171a-Brassica_rapa-21664	100	100	1	131	71	463	1.43E-90
BraA03006359	PUT-171a-Brassica_rapa-43719	72	98	38	62	512	438	2.02E-09
BraA03006360	PUT-171a-Brassica_rapa-43719	76	99	109	125	1011	1061	1.91E-08
BraA04000985	PUT-171a-Brassica_rapa-38377	71	100	1	76	211	471	4.02E-17
BraA04000985	PUT-171a-Brassica_rapa-38377	96	100	25	51	315	395	3.43E-11
BraA04001583	PUT-171a-Brassica_rapa-18109	92	92	2	13	228	263	2.31E-20
BraA04001674	PUT-171a-Brassica_rapa-15821	86	100	1	56	1088	921	4.11E-25
BraA04002060	PUT-171a-Brassica_rapa-17301	72	78	1	72	211	2	4.33E-26
BraA04002063	PUT-171a-Brassica_rapa-17301	72	78	1	72	211	2	4.33E-26
BraA04003089	PUT-171a-Brassica_rapa-26190	100	75	16	61	755	618	4.52E-25
BraA05002292	PUT-171a-Brassica_rapa-5019	70	96	1	105	539	985	1.84E-64
BraA05002383	PUT-171a-Brassica_rapa-30964	76	91	40	77	636	749	5.00E-22
BraA05002383	PUT-171a-Brassica_rapa-30964	71	91	1	35	517	621	5.00E-22
BraA05002383	PUT-171a-Brassica_rapa-30964	86	91	82	95	761	802	5.00E-22
BraA05002383	PUT-171a-Brassica_rapa-3975	92	100	41	77	192	82	1.87E-17
BraA05002386	PUT-171a-Brassica_rapa-3975	88	100	1	25	13	87	2.46E-08
BraA05002698	PUT-171a-Brassica_rapa-28823	80	78	12	31	415	474	1.40E-05
BraA06000802	PUT-171a-Brassica_rapa-37927	100	100	1	108	542	865	8.45E-29
BraA06001127	PUT-171a-Brassica_rapa-29270	96	100	9	54	161	298	1.77E-09

BraA06001127	PUT-171a-Brassica_rapa-29270	100	100	1	19	123	179	7.61E-05
BraA06001127	PUT-171a-Brassica_rapa-2695	84	80	7	49	258	386	4.63E-05
BraA06001678	PUT-171a-Brassica_rapa-20908	100	72	1	46	161	24	9.89E-25
BraA06001680	PUT-171a-Brassica_rapa-20908	100	72	1	46	161	24	9.89E-25
BraA06002266	PUT-171a-Brassica_rapa-39669	100	100	1	58	242	415	5.74E-36
BraA06003518	PUT-171a-Brassica_rapa-5133	78	70	22	71	412	263	5.19E-20
BraA06003571	PUT-171a-Brassica_rapa-36962	99	71	1	250	79	828	0
BraA06003586	PUT-171a-Brassica_rapa-19143	100	75	18	109	151	426	4.73E-64
BraA06004921	PUT-171a-Brassica_rapa-8594	98	76	1	40	136	255	5.92E-27
BraA06004921	PUT-171a-Brassica_rapa-9077	98	76	1	40	130	249	6.16E-27
BraA07000468	PUT-171a-Brassica_rapa-44096	100	96	1	70	440	649	1.54E-44
BraA07000615	PUT-171a-Brassica_rapa-145599	100	100	1	52	328	173	5.07E-19
BraA07000755	PUT-171a-Brassica_rapa-2970	93	100	1	121	956	591	7.44E-73
BraA07001366	PUT-171a-Brassica_rapa-2508	100	74	6	69	61	252	5.67E-34
BraA07001980	PUT-171a-Brassica_rapa-10526	76	84	1	59	40	216	6.56E-32
BraA07001980	PUT-171a-Brassica_rapa-10526	75	84	60	99	216	335	6.56E-32
BraA08000151	PUT-171a-Brassica_rapa-25434	100	100	1	132	707	312	8.97E-93
BraA08000151	PUT-171a-Brassica_rapa-25406	98	86	19	132	671	330	4.47E-75
BraA08000151	PUT-171a-Brassica_rapa-31475	74	75	15	82	268	65	5.36E-29
BraA08001993	PUT-171a-Brassica_rapa-8737	95	83	1	65	369	563	4.31E-36
BraA08003083	PUT-171a-Brassica_rapa-40152	100	100	1	111	587	255	4.19E-74
BraA09000145	PUT-171a-Brassica_rapa-9077	100	100	1	58	130	303	1.97E-36
BraA09000145	PUT-171a-Brassica_rapa-8594	100	100	1	58	136	309	7.75E-36
BraA09001463	PUT-171a-Brassica_rapa-145599	100	100	1	52	328	173	5.07E-19
BraA09001629	PUT-171a-Brassica_rapa-36430	95	100	1	59	22	198	1.76E-25
BraA09003321	PUT-171a-Brassica_rapa-38377	91	77	14	57	247	116	1.36E-11

BraA09003322	PUT-171a-Brassica_rapa-38377	95	100	1	63	211	399	2.44E-24
BraA09003504	PUT-171a-Brassica_rapa-33928	98	76	16	62	172	312	2.28E-25
BraA09004141	PUT-171a-Brassica_rapa-28135	100	100	1	62	33	218	3.90E-39
BraA09006583	PUT-171a-Brassica_rapa-36625	100	100	1	79	261	25	6.42E-29
BraA10000875	PUT-171a-Brassica_rapa-9845602	100	100	1	67	851	1051	8.44E-39
BraA10003123	PUT-171a-Brassica_rapa-9690	93	87	42	132	314	586	2.04E-57
BraA10003123	PUT-171a-Brassica_rapa-9690	100	87	18	44	169	249	8.62E-12
BraSca000413	PUT-171a-Brassica_rapa-4535	100	72	1	72	423	208	6.85E-45
BraSca001227	PUT-171a-Brassica_rapa-37124	100	100	1	47	13	153	9.09E-26
BraSca001227	PUT-171a-Brassica_rapa-37124	100	100	47	66	444	503	3.00E-06

Table S5. Expression patterns of BSGs in different Chinese cabbage tissues. FPKM—fragments per kilo bases of exons for per million mapped reads.

	Flower	Leaf1	Leaf2	Root1	Root2	Silique	Stem	Total
With expression abundance (FPKM > 2)								
Number of BSGs (%)	116 (15.41)	62 (8.23)	86 (11.42)	90 (11.95)	119 (15.80)	154 (20.45)	126 (16.74)	753 (100.00)
With high expression abundance (FPKM > 50)								
Number of BSGs (%)	18 (17.14)	8 (7.62)	8 (7.62)	14 (13.33)	13 (12.38)	28 (26.67)	16 (15.24)	105 (100.00)
With tissue-specific expression (FPKM > 2)								
Number of BSGs (%)	16 (17.02)	0 (0.00)	4 (4.26)	4 (4.26)	6 (6.38)	55 (58.51)	9 (9.57)	94 (100.00)

Table S6. Expression patterns of BSGs after Pb treatment in Chinese cabbage.

	0 hai	12 hai	72 hai	96 hai	Total
With expression abundance (FPKM > 2)					
Number of BSGs (%)	119 (24.79)	127 (26.46)	114 (23.75)	120 (25.00)	480 (100.00)
With high expression abundance (FPKM > 50)					
Number of BSGs (%)	18 (27.27)	13 (19.70)	15 (22.73)	20 (30.30)	66 (100.00)
With period-specific expression (FPKM > 2)					
Number of BSGs (%)	9 (28.13)	12 (37.50)	8 (25.00)	3 (9.37)	32 (100.00)

Table S7. Primer sequences used for BSGs validation and expression pattern analyses.

Gene IDs	Validation primer pairs		Semi-qPCR and qRT-PCR primer pairs	
	Sequences of primer F1 (5'-3')	Sequences of primer R1 (5'-3')	Sequences of primer F2 (5'-3')	Sequences of primer R2 (5'-3')
**BraA03000262**	ATGAGGGAGACCGAGGCGT	CTAAATGCAAAACCTGTCATTG	The same as F1	The same as R1
**BraSca000334**	The same as BraA03000262 F1	The same as BraA03000262 R1	The same as BraA03000262 F1	The same as BraA03000262 R1
**BraA04003089**	ATGAATCAAAAGGCTAGGCT	TCAGGCGTCGGAGATCCGGT	TCAAAAGGCTAGGCTGAAGCA	GAGATCCGGTGGTGTTCCTC
**BraA05003308**	ATGGGTTTCTCTCCAACTC	TCAGAGCACTACTCGAAACC	The same as F1	The same as R1
**BraA09004053**	The same as BraA05003308 F1	The same as BraA05003308 R1	The same as BraA05003308 F1	The same as BraA05003308 R1
**BraSca000445**	The same as BraA05003308 F1	The same as BraA05003308 R1	The same as BraA05003308 F1	The same as BraA05003308 R1
**BraA05003841**	ATGGCGACTCTTCCCTTTGCG	TTATACAGACAGAAGACTACA	GTGGCTCTACTCGACGGAAC	AGGCACCGATAGAAAGGTCG
**BraSca001097**	The same as BraA05003841 F1	The same as BraA05003841 R1	The same as BraA05003841 F2	The same as BraA05003841 R2
**BraA07000615**	TGCAGCGGCGGAATTGTA	ACTTGTTACCCCCTCCTGGT	The same as F1	The same as R1
**BraA09001463**	The same as BraA07000615 F1	The same as BraA07000615 R1	The same as BraA07000615 F1	The same as BraA07000615 R1
**BraA09001629**	TGGAAGGTATGTTTTTGCTCAAATC	ACAAACCACTGACTGCCAGA	AGAAAAAGAGATTCACCGCCG	GCCAAAATCTGGTGTCCTCG
**BraA09002618**	ATGGGAAAGAAAGAGACAGTTGG	TCAGAACAAGGAGGACCCGCG	The same as F1	The same as R1
**BraSca000945**	The same as BraA09002618 F1	The same as BraA09002618 R1	The same as BraA09002618 F1	The same as BraA09002618 R1
**BraSca001287**	The same as BraA09002618 F1	The same as BraA09002618 R1	The same as BraA09002618 F1	The same as BraA09002618 R1
**BraA09003195**	ATGCGAGAATCGGTGCGAGA	CTACTTGGACCGCTGATTCTTGT	The same as F1	The same as R1
**BraSca001271**	The same as BraA09003195 F1	The same as BraA09003195 R1	The same as BraA09003195 F1	The same as BraA09003195 R1
**BraA10000178**	ATGGGACTTTGGCACAGAG	TTAAAAGAAGAGAGCAAAGAGT	TGCTCCACGGTGCTAATG	AGAGCAAAGAGTAGCTGACAAG
**BraA10000249**	ATGTCAGATCGAGGATCTGTT	TCACCGATAAGAAGCCAAA	The same as F1	The same as R1
**BraA10000359**	ATGCAGAAAACTCTGCAGG	TTAAGCTCTAACAAATTGGTCT	GTGTGGAAGCTCAGCGTATAA	CTACCACGAGCTTGCCTATTT
**BraA10000477**	ATGGTAAAATATCTTTGAGCATA	TCAGAAAGAAATCACAAGAAAAC	AGCAATCGACGGTTTCTCTC	ACTCGAAGACGGCACTAGA
**BraA10000717**	ATGGTTCAAGAACAAGAGATGA	TTAGACTCTCCTAGCACAACCA	The same as F1	The same as R1
**BraA10000759**	ATGAGGCTAAGGGATCCTGA	TCAGTTTGCTAGAAGAACCTGC	CCCGCCACCTTCTTCTTATT	GGAAAGGATGTGAGGAGTAACC

**BraA10000785**	ATGTCAGGTGCTTCAACTGTTCA	TCAGGAAGGATCTGCCCGC	ATGTCAGGTGCTTCAACTGT	The same as R1
**BraA10001918**	ATGCGCACCCGTCGAGAAC	TTAGGCTTGTCTGCTTAAACC	The same as F1	The same as R1
**BraA10001919**	ATGAATTCAAACCAATCCATG	CTACCGACCCGAGAGGATC	GGGAATAGAGGAAAGCAAGTGT	GACGACGGAGAAGATGTCATAAG
**BraA10002884**	ATGAAACAAAGACTAAGACA	TCAACGACGGAACCCACC	The same as F1	The same as R1
**BraSca000020**	ATGCGCAGTCGAGCCACT	TTATGAGCAGAACAATCTGATG	The same as F1	The same as R1
**BraSca000143**	ATGGAGCCAATGGTGTGGT	CTAATCATCGCAGAGAGACTGG	The same as F1	The same as R1
**BraSca000146**	ATGAAGCATCGATCGACACT	TTAAGGTTTATGCCGATCAA	GAAGCGACGATCCACAAAGA	CCATGGGAAGGCCATGATAC
**BraSca000160**	ATGGAGTGTGTTGGTGCTGT	TCAGAAATTTGAATCAGTGG	The same as F1	The same as R1
**BraSca000205**	ATGAAGCCACTGATAATTGG	TTAATCACAAATATCTTCGG	The same as F1	The same as R1
**BraSca000216**	ATGATATCTGTTATAGTTGG	CTATCTCTTTTTGAACTTGT	TGAAGTTGTTGGAGGAGTTGAG	CCCGGAAAGGTCACGATAATAC
**BraSca000270**	ATGATAACACAATGGGAAGA	TCATAGGGACATCTGAAATGAT	The same as F1	The same as R1
**BraSca000289**	ATGGATCCTCCCAGATCCGGTC	TCAGTTGGCGCCTGCTGAAG	The same as F1	The same as R1
**BraSca000335**	ATGTCGAGCAATTTGGAGG	TCAAATGGTTCTGATAAATCGA	The same as F1	The same as R1
**BraSca000346**	ATGGTAGTGTCTGGTCTGTTA	TCACAATCCCCTCAACAGT	GGGGAGGATGTTGCGAATCT	ACAATCCCCTCAACAGTGGC
**BraSca000367**	ATGATCTATCCTCTAGAGAAAG	TCAGGGATCCTCTGTTGTC	The same as F1	The same as R1
**BraSca000423**	ATGCCCGCTCTAATGTGCG	CTATTTGCCGATCGACTGC	CTTGTGGCATTGTGAGGTTTG	TCACCAAAGGAACCGATACAC
**BraSca000441**	ATGTCAAGCGACAACAATG	TCATGGGTTGTGACTTCTT	The same as F1	The same as R1
**BraSca000476**	ATGAAGTTACAACCTTACTTCC	TTATCCTCCCGAAACCTGG	The same as F1	The same as R1
**BraSca000502**	ATGCTGGAGTTGATGAAGATCA	TCACGTCGGTATCTGCTGAT	The same as F1	The same as R1
**BraSca000544**	ATGGATCGAAACTTCATGTA	CTATTCTGACAATAGATTTTTTC	GTCTATGTTCAGTGTTTGCTTCAG	AATATCTGCGAGTCGAAGCC
**BraSca000553**	ATGAAGGTGGTCAAGGCAGA	TCACTTCCACCACGTCTCAT	The same as F1	The same as R1
**BraSca000578**	ATGATCTTGTGGTCTTTCGG	TTAGTACTTACCCTGGGTTGTA	TTCGACTAATGCTCGCCATAC	ACCGCAGAGTCTATTCAAACC
**BraSca000608**	ATGACATCTCTTCTTCATGACGC	CTAAAGCCAAATTAAACCTAATG	The same as F1	The same as R1
**BraSca000627**	ATGGTATATCTTTTGGTAACAC	CTACAACTGCAAAAGCATGTAC	AGGGCATTATAGGCAGTTATCC	CCTCTTCCAGGCTTTATCCTATC
**BraSca000661**	ATGCCCGGGAGTTTGTTCA	TTACTCTCCGGGTTCTTCCC	The same as F1	The same as R1
**BraSca000870**	ATGACAATAGCAGCGCCGG	TCAATCCGACAGAGAGAGGCTC	CCTACAACGTGCGCCTATC	TCGATTTGGTGGTTGGTCTT

**BraSca001039**	ATGGATGTTCTCAAATTGCATA	TTAATCTTGATCAGAATCGAAA	The same as F1	The same as R1
**BraSca001087**	ATGGCTGACGGCCCTAACTT	CTAGCCCGCTCTGCCTTCAA	The same as F1	The same as R1
**BraSca001249**	ATGATGCTACTCACTCGAGACAA	CTAGCTCCACCACGATGGCG	The same as F1	The same as R1
**BraSca001291**	ATGTTTTGGGCGGTTTCCTC	TTACAGGAGCAGTTCCTGGAC	The same as F1	The same as R1
**BraA09001316**	ATGAGCTTCGGAGAACCTGC	CTATTCAGAGAATCTCTTTA	/	/
**BraA09001317**	ATGGGTCTACTCCCTCAGCG	CTAGCCGCCAATTGGTTGGT	/	/
**BraA10000028**	ATGATTTCCACTTTAAAGCTAA	TCACCACATGAATCTTTGAAAC	/	/
**BraA10000827**	ATGCAGATCGCATGTCGGCATA	TTAGATGATATAGCTAATGTGGG	/	/
**BraA10001420**	ATGGATTTATCGTTTATCATAC	TCAAGCAAAAGAAAACTCAAACGC	/	/
**BraA10002472**	ATGTATGATGTATCTCAGGTG	TCACAGCATTTTGAGGCAGT	/	/
**BraA10002976**	ATGGTTGTGGTGGTGGAGGA	TCATGATACGAGCCACATATAG	/	/
**BraSca000038**	ATGCTTCCCGAATCCCACATTGA	TCATGGATGGAGAACCAGATC	/	/
**BraSca000039**	The same as BraSca000038 F1	The same as BraSca000038 R1	/	/
**BraSca000068**	ATGAGAGTTTCGTTTGTTTT	CTAATCAGGCCTTAGAAACA	/	/
**BraSca000088**	ATGTATGATGGCGATCAATTG	TTATTTTCTTCCCATTTCCA	/	/
**BraSca000214**	ATGCCGCTTATTCAATCTACTGA	CTAACTCGGAGACGAACCGT	/	/
**BraSca000215**	ATGACTTGTCTTGCTAAGTA	CTACATGAGGTACGGGATGTCG	/	/
**BraSca000222**	ATGCCGGTTAACGGTGGTGAGAT	TCAGAAAAGTCTAAAGCCTCGA	/	/
**BraSca000301**	ATGCCCAAGCTCGGTCGGTCGAC	CTACATATGCGAGAGAAGCGCC	/	/
**BraSca000302**	ATGGGTCGTAAAAACCGATA	TCACAAACGCACTGGTCTGG	/	/
**BraSca000303**	ATGAGCGGGAACTCATGTTTA	TCAACTCATCTTACCCATAA	/	/
**BraSca000480**	ATGCTGCCTCCTTCTAAGCTG	TCAAAAAACTCGAGTACATTCA	/	/
**BraSca000481**	ATGGCGAGCGGATCCAGCGC	CTAGTTCGGCCATCCACCGAGCT	/	/
**BraSca000527**	ATGTATGCTGTTCAGTCGCGCGGTG	CTACCACCAGACACATACTCC	/	/
**BraSca000623**	ATGGTGACGGTTCTGTTTGA	TCAACCGAAAGGAAGGAGGACAA	/	/
**BraSca000630**	ATGGTGTCCCCAACAATGCG	CTATGCCTGAGTAGTTCGTATTC	/	/

**BraSca000632**	ATGGCGCTCGCGAGCTTCCACGCCT	TCAGAGAGGAGCAACGAGACAAA	/	/
**BraSca000634**	ATGTCCTCGCCCGCCCCCGC	TTAGCCCTCTGTGCCCTGGCGG	/	/
**BraSca000635**	ATGGCTCGCCGCACTGCCCCCCGCT	TCAGGGGAGTGGAGCGGGAGAACC	/	/
**BraSca000636**	ATGCACTTCCATACACTCTTG	TCAGGGGAGGTGTGGGGAGAG	/	/
**BraSca000642**	ATGCCAGAGTGCGGCCAGTGGGT	TCACGTTCTCCCCAACTCGCCCGCC	/	/
**BraSca000643**	ATGACTTCCTGTTATCCCTTGGCG	TCAAGTTTGACCAGGCGTGC	/	/
**BraSca000647**	ATGACTCATGATTTGAATCGGCCG	CTAGGTTTCATCCCGGGGCCT	/	/
**BraSca000648**	ATGGAGGAAGTAGTGAATAAACT	TTATAACCGTCTGAAAGCCTTAT	/	/
**BraSca000686**	ATGTACTACATTCTTCATCGGCGA	TTAGACAGGAAAAGAACCATCTGA	/	/
**BraSca000687**	ATGGCGCAACAATTAGAAGAAA	TTAGAAGTCTGTGCTTGAGCGCT	/	/
**BraSca000726**	ATGCATATTTCGTACGATGA	CTAGAAGCTAACATATCTAG	/	/
**BraSca000729**	ATGGCGTCGCTGCAGACTTAT	TCATACTATCCTACCGTGGCCA	/	/
**BraSca000752**	ATGGGTGGGTGGAATATGGCTG	TCACGCCGTCACGAGTGCGG	/	/
**BraSca000754**	The same as BraSca000752 F1	The same as BraSca000752 R1	/	/
**BraSca000757**	ATGCATAGTAACGGAATAAAGACA	TCAGGACCGTCTGATATGTGTAT	/	/
**BraSca000758**	ATGTGTGTCCGTGAGTGTCC	CTAACGGAAACACATGGACA	/	/
**BraSca000768**	ATGACGATTTCGTCTCCGCA	TCAGGATGCCTGAGCCCGTGG	/	/
**BraSca000811**	ATGGCCTTGTCCCAAGCCCC	TCACTGAAAGAAAGATCTGAA	/	/
**BraSca000840**	ATGGGAACCATGGCTCAAGA	TTAGCGCATGGAAATTGAATC	/	/
**BraSca000842**	ATGGCGGCCGTATCCGCTATT	CTAAGGTTCACAGGGCAATCCA	/	/
**BraSca000849**	ATGACTGTGCTACAGTACTA	TTAGAGTAAGACCCCGGCTG	/	/
**BraSca000878**	ATGACGGAGAATGAGTCGAA	TCATGGACCGCCGCACGATT	/	/
**BraSca000879**	ATGAACGACAGCCGACGGAAC	CTACTGGTAGCATTCTAACA	/	/
**BraSca000880**	ATGAAGGCGGCACTTGGGCA	CTACGTGTTGGTCCGTTCTG	/	/
**BraSca000881**	ATGGAAGATAATGAGTCGCA	TTAAAGAACATGTGGCTTCC	/	/
**BraSca000882**	ATGGCTAGCCTGAACTGGAG	TCAGCTGTGAGTCAGAAGAT	/	/

**BraSca000883**	ATGTGGAAGTTGATAACATAC	TCAACGAACACCACATGGAC	/	/
**BraSca000884**	ATGAAAGATGTGGTGAGAGA	CTACCTGACTGTGTCCCTCT	/	/
**BraSca000902**	ATGCGTATTAGCAGAAAATACA	CTAATAACCTTGCTTCTGCT	/	/
**BraSca000910**	ATGGATGCGAAGAAGCTTAGC	TCAACTACTACGGCCTGGTT	/	/
**BraSca000914**	ATGTCTCATACTGATCTGAA	TCAGGTTCCATCCACGGGGA	/	/
**BraSca000915**	ATGGCGTTCATACCGCCCAG	TTACTGGTGTAAAGCATGGG	/	/
**BraSca000929**	ATGGTAGCCACGACCATCG	TTATTCACGCCCGGACAGAAC	/	/
**BraSca000930**	ATGTACAGGCTCACTCTTTCTC	CTATTCGAATGTGTCTTCTCTGTA	/	/
**BraSca000932**	ATGAAGGATAGGCATTTTTTCC	CTAACGGATTGCCATAAGGACGT	/	/
**BraSca000933**	ATGCGAACCGGCGACGCCCAC	TCATTGTTTATAGATCTCTTC	/	/
**BraSca000936**	ATGCTCATAGTAGGACATTTT	TTAAATGGATCTCACTTTTG	/	/
**BraSca001006**	ATGGGTGGTCTCGATAGTAA	CTATCCCGTCCCATCGTGGCG	/	/
**BraSca001077**	ATGCCGCGCATGTCCACAAGA	TTACTCTACTCCTATGGCAATTT	/	/
**BraSca001082**	ATGCTAGGGAGATGCAGCCC	CTACCCGACTAGTCCAGGCT	/	/
**BraSca001101**	ATGAGACCGAACCCGCGAAC	TTATGGATAAAGGAATGATGGA	/	/
**BraSca001121**	ATGCCGGGCCGCCGAGGCCGCG	CTACGTCCTCGGGGCGTTGTT	/	/
**BraSca001125**	ATGGTTAGCGAGTCTCCCGGC	TCACTCCATCTCAGCCCCTACTA	/	/
**BraSca001133**	ATGGTTCCTTTGTATACATCAC	TCACTTTTCGTGTGCGCACCCGGA	/	/
**BraSca001134**	ATGGCTCTTTTTGAAGGAAGAC	TCAGGTCCTCAGCGGCTTACT	/	/
**BraSca001135**	ATGTATGAGCCAGAGTCATCGA	TCAGATTGAAACGGCTCCGG	/	/
**BraSca001145**	ATGGTACGCTGGGAGGGGCC	CTAGCCTGATAATCTCGCGC	/	/
**BraSca001146**	ATGGAACCGGTATCTAACTT	TCAGCCAGCATGCATTGCCAGCA	/	/
**BraSca001150**	ATGGACGATGATATAGTCTC	TCAGCGTACTGGCGCTGAGC	/	/
**BraSca001191**	ATGTACAAGAGTGCGATCACGGC	CTAGCCTTCAATCAGTGAGC	/	/
**BraSca001193**	ATGCTGAACACGGGAGCGGAA	TCATATCTCTATGCGTTTCAC	/	/
**BraSca001199**	ATGTGTGCTGTAGGCGCGC	TCAGCGGCACAGTCGAAGCA	/	/

**BraSca001208**	ATGAGGTTGGGAAAGAGGAAGG	TCACATCAAAGCATAAACACCAA	/	/
**BraSca001257**	ATGAAACCACCGATGTGGAAG	CTAACCCTCTATAAATACCGCT	/	/
**BraSca001267**	ATGAAGATAGCCGTTTGTACAC	CTAGGTGTACTGATACAAATC	/	/
**BraSca001275**	ATGATCAATAAAACCCCACGTAC	TTAACACCGACCGTCATTTA	/	/
**BraSca001278**	ATGAACATGAGACCATGTA	TCAGTAGTATATGAGGCAGCAT	/	/
**BraSca001288**	ATGACAGAGCAACTGTCGAC	TCAGGGTCCACGAAGCTTAT	/	/
**BraSca001294**	ATGGTACATTTCGACGCTGGG	TTAACTTGGTGCAGCATTGA	/	/
**BraSca001297**	ATGTTATTCATTCGCTTTTAC	TTAAAGTACGTTTAGCGACAAG	/	/
**BraSca001299**	ATGAAGAATATATGTAGGT	TCAGTCAGGTGACGCATGGG	/	/
**BraSca001301**	ATGAAAGACGTCCGGGGAAG	CTACCCTTCCCCCCTCCCCTC	/	/
**BraSca001302**	ATGGGGCAAGAGGAGTGTACG	TCATTTTTTCTCCTCCCTGT	/	/
**BraSca001303**	ATGGGAGCAGGAGCAAAGGA	TTAGAGCTTCCTCTCTCCTG	/	/
**BraSca001304**	ATGGAACCGACAAATGACAGG	CTACGCTGCCAGCTTCACCC	/	/
**BraSca001305**	ATGCGAAGAACAAGCGAATT	TCATTTTGGCAAATGTGAAATG	/	/
**BraSca001307**	ATGATATCGAATCATATCTCC	TCAGGTCAGCCAGCCACTGGAG	/	/
**BraSca001309**	ATGAAGTCGTCGCGTCGTTACT	CTAGCGACGGTTGTGAGGAC	/	/
